# Safety and efficacy of COVID-19 vaccination in the Chinese population with pulmonary lymphangioleiomyomatosis: a single-center retrospective study

**DOI:** 10.1186/s13023-024-03260-4

**Published:** 2024-07-03

**Authors:** Weilin Wang, Jingdong Zhou, Xuetao Kong, Yixuan Wang, Qixian Wu, Xiaoqing Gong, Jingye Tai, Yingxin Pan, Hongyuan Huang, Zhen Zhao, Mei Jiang, Jie Liu

**Affiliations:** 1 Nanshan College, Guangzhou Medical University, Guangzhou, China; 2 School of Public Health, Guangzhou Medical University, Guangzhou, China; 3 First Clinical College, Guangzhou Medical University, Guangzhou, China; 4 Department of Respiratory and Critical Care Medicine, National Center for Respiratory Medicine, National Clinical Research Center for Respiratory Disease, Guangzhou Institute of Respiratory Health, State Key Laboratory of Respiratory Diseases, National Clinical Medical Research Center for Respiratory Diseases, The First Affiliated Hospital of Guangzhou Medical University, Guangzhou, China

**Keywords:** COVID-19, Lymphangioleiomyomatosis, mTOR inhibitors, Omicron variant, Vaccine

## Abstract

**Background:**

The safety and efficacy of vaccination against coronavirus disease 2019 (COVID-19) in patients with lymphangioleiomyomatosis (LAM) is still unclear. This study investigates COVID-19 vaccine hesitancy, vaccine safety and efficacy, and COVID-19 symptoms in LAM patients.

**Results:**

In total, 181 LAM patients and 143 healthy individuals responded to the questionnaire. The vaccination rate of LAM patients was 77.34%, and 15.7% of vaccinated LAM patients experienced adverse events. Vaccination decreased the risk of LAM patients developing anorexia [OR: 0.17, 95% CI: (0.07, 0.43)], myalgia [OR: 0.34, 95% CI: (0.13, 0.84)], and ageusia [OR: 0.34, 95% CI: (0.14, 0.84)]. In LAM patients, a use of mTOR inhibitors reduced the risk of developing symptoms during COVID-19, including fatigue [OR: 0.18, 95% CI: (0.03, 0.95)], anorexia [OR: 0.30, 95% CI: (0.09, 0.96)], and ageusia [OR: 0.20, 95% CI: (0.06, 0.67)].

**Conclusions:**

Vaccination rates in the LAM population were lower than those in the general population, as 22.7% (41/181) of LAM patients had hesitations regarding the COVID-19 vaccine. However, the safety of COVID-19 vaccination in the LAM cohort was comparable to the healthy population, and COVID-19 vaccination decreased the incidence of COVID-19 symptoms in LAM patients. In addition, mTOR inhibitors seem not to determine a greater risk of complications in patients with LAM during COVID-19.

**Supplementary Information:**

The online version contains supplementary material available at 10.1186/s13023-024-03260-4.

## Background

Lymphangioleiomyomatosis (LAM) is a rare disease that results in the widespread cystic degeneration of lung tissue. It is classified as a low-grade malignant neoplasm that can metastasize and invade surrounding tissue. Recent studies have estimated that the incidence of LAM in the general population is between 20.9 and 26.04 cases per million adult women [1]. Based on genetic features, it can be divided into two clinical subtypes: (i) sporadic LAM (S-LAM) and (ii) tuberous sclerosis-associated pulmonary LAM (TSC-LAM) [2]. Tuberous sclerosis complex (TSC) is an autosomal dominant disease that arises from mutations in the TSC1 or TSC2 genes, with a prevalence of approximately 1 in 5000. It is characterized by nonmalignant malformations of several organs, such as the brain, skin, kidneys, lungs, heart, and retina [3]. S-LAM patients mainly present with recurrent pneumothorax or chylothorax with progressively worsening dyspnea, and approximately 29% of them also presented with renal angiomyolipoma [4, 5]. In 2008 and 2011, Bissler and McCormack demonstrated that the use of sirolimus, a mammalian target of rapamycin (mTOR) inhibitors, stabilizes LAM patients by improving lung function and exercise tolerance and decreasing renal angiomyolipoma volume [6, 7]. Sirolimus has been approved for treating LAM and TSC-related conditions in the United States and Japan [8]. The first Chinese expert consensus on using sirolimus to treat LAM was released in 2019 [9].

The Omicron variant of severe acute respiratory syndrome coronavirus 2 (SARS-CoV-2) was first detected in South Africa in late November 2021 and has rapidly become the most prevalent variant worldwide [10, 11]. This variant is highly infectious, with lower rates of severe illness and death than the Delta variant, representing a new challenge to global public healthcare [12]. Established epidemiological data indicate that populations with comorbidities and those who are unvaccinated face higher risks of severe illness [13]. However, the safety profile of the COVID-19 vaccine among LAM patients is unclear. Furthermore, the specificity of the vaccine’s protective effect remains to be determined. There is an extreme lack of clinical reports related to COVID-19 vaccination and infection with the Omicron variant in specific populations, such as patients with the rare respiratory disease LAM. Although LAM has been shown to increase hospitalization rates for COVID-19, most of the patients in the studies were unvaccinated or infected with SARS-CoV-2 prior to the emergence of the Delta variant [14]. There are no reports on the protective effect of LAM in patients who received the COVID-19 vaccination following infection with the Omicron variant. Furthermore, Baldi et al. found that in some LAM patients with SARS-CoV-2 infection, sirolimus treatment may be beneficial [14]. However, this has not been reported during the Omicron variant pandemic.

We conducted a retrospective study at a single center to evaluate the vaccination of LAM patients, the reasons for vaccine hesitancy, vaccine safety and efficacy, and the potential impact of mTOR inhibitors in LAM patients infected with the SARS-CoV-2 Omicron variant.

## Methods

### Study design and population

**Fig. 1:**
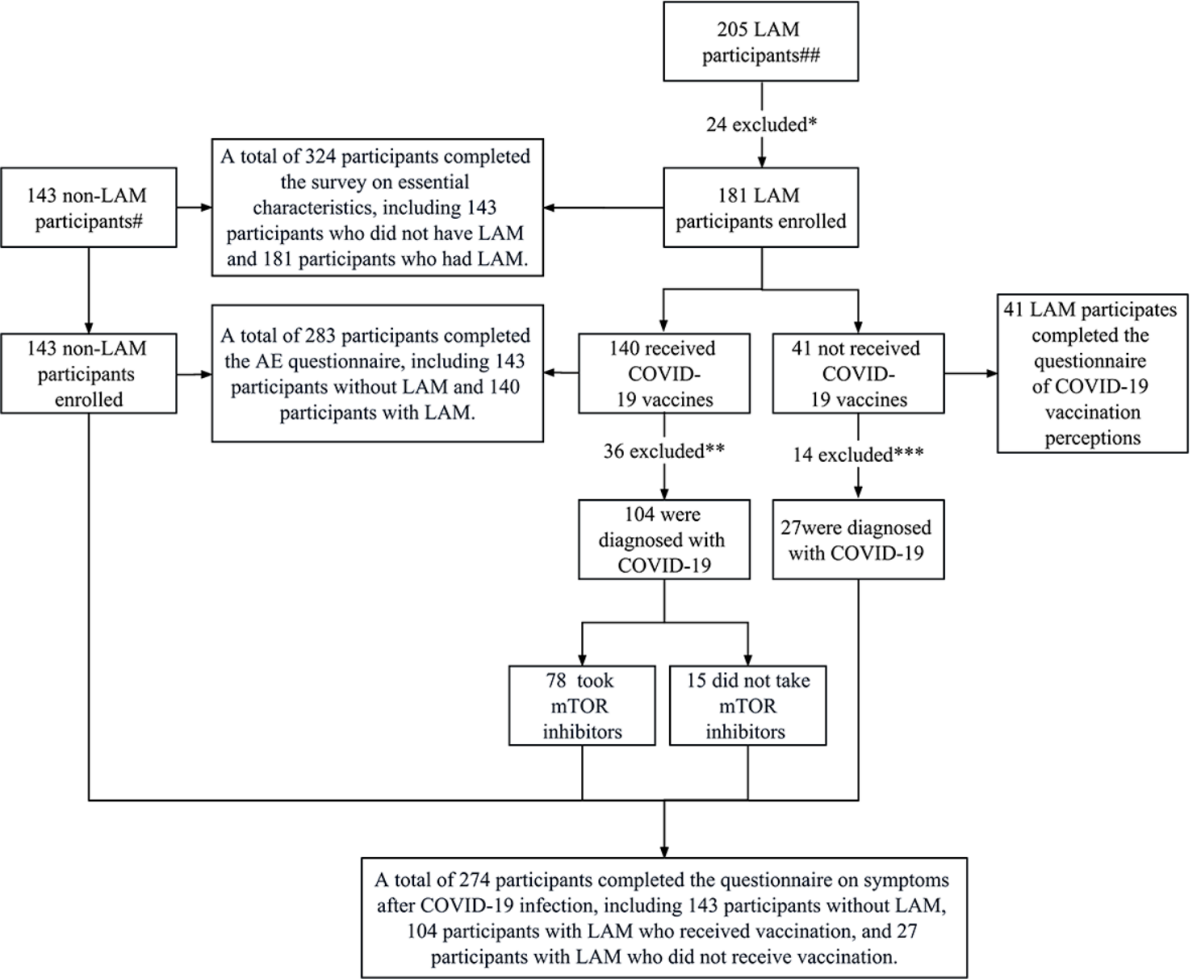
Study profile *Exclusion criteria: Participants who fail to answer basic questions, provide incorrect answers, or refuse to participate (*n* = 24) ** Exclusion: Participants who have not been diagnosed with COVID-19 (*n* = 36) *** Exclusion: Participants who have not been diagnosed with COVID-19 (*n* = 14)

This retrospective study of 181 Chinese LAM patients followed the STROBE guidelines [15] and was initiated by the Center for Rare Respiratory Diseases of the First Affiliated Hospital of Guangzhou Medical University. Questionnaires were collected through the outpatient clinic and the chat group of LAM patients, thus enrolling 181 LAM patients from various provinces and cities of China. In addition, 143 healthy controls were recruited from each province and city. The inclusion criteria for LAM patients were as follows: (1) aged ≥ 18 years, (2) diagnosed with LAM, (3) female and not pregnant, and (4) willing and capable of completing the questionnaire. The diagnosis of LAM was based on the 2017 American Thoracic Society/Japan Respiratory Society clinical guidelines and the 2010 European Society for Respiratory Medicine guidelines. The inclusion criteria for the healthy control population were as follows: (1) aged ≥ 18 years, (2) female and not pregnant, (3) no history of LAM or any other respiratory disease, and (4) willing and capable of completing the questionnaire. In this study, the definition of SARS-CoV-2 infection was as follows: (1) a positive nucleic-acid or antigen test or (2) a history of contact with a patient during the Omicron variant epidemic in China, accompanied by the presence of typical symptoms [16, 17]. Questionnaires that were not completely filled out or contained inconsistent answers were excluded. Figure 1 depicts a flow chart of this study.

### Data collection

Data were gathered from December 1, 2022, to January 20, 2023, utilizing online questionnaires, telephone follow-ups, and hospital outpatient visits. We collected the following four primary components:

 (1) Baseline data were collected for LAM patients and healthy controls, including age, body mass index (BMI), comorbidities, smoking status, and regular use of mTOR inhibitors. In addition, we collected daily oxygen saturation and dose of mTOR inhibitors used regularly to assess the condition of LAM patients.

 (2) The vaccination status of LAM patients and healthy controls was documented for vaccine reactions (e.g., redness, swelling, induration, and pain at the site of vaccination, fever, fatigue, nausea, vomiting, headache, myalgia, diarrhea, and arthralgia) and the use of mTOR inhibitors during the vaccination period. The criteria for vaccination required at least one dose of vaccine to be received by the end of the follow-up visit. The adverse reaction classification of vaccines is referred to the document of the China Food and Drug Administration [18]. Adverse reactions to vaccines were defined as those occurring within seven days of each vaccination.

 (3) For unvaccinated patients, reasons for not receiving the vaccine included “concerns about the safety of the vaccine,” “concerns that vaccine may exacerbate LAM symptoms,” “concerns about interactions between the vaccine and LAM treatment medications,” “do not think the vaccine is necessary,” “my doctor does not recommend the vaccine for LAM patients,” and “just waiting.”

 (4) This study compared the occurrence and duration of COVID-19 symptoms between LAM patients and healthy controls. We also investigated the exacerbation of symptoms in LAM patients during the COVID-19 period.

### Preparation and revision of the questionnaire

The items in the questionnaire were developed from February to December 2022 based on expert opinions, relevant literature, and a pre-survey. We formulated simple easy-to-understand questions that explained specialized terms to minimize measurement error. Online surveys could use automated skip logic to bypass questions that do not require responses, aiming to reduce logical discrepancies. Furthermore, to minimize potential missing data, the questionnaire could only be submitted after the informed consent form had been signed and the key questions had been answered. The survey data were exported as an Excel file from the online platform, and duplicates were removed based on the patient’s contact information and date of birth. When duplicates were found, only the earliest post-infection record was retained to obtain the questionnaire information for that individual. Questions regarding demographics, COVID-19 vaccination, “Reasons for COVID-19 Vaccine Hesitation,” “Duration of COVID-19 Symptoms,” and “Administration of mTOR” were not considered for the reliability evaluation. Cronbach’s alpha coefficient was employed to assess the reliability of the questionnaire, and its obtained value was 0.806. Moreover, we developed the questionnaire based on the guidelines prescribed and consulted experts to gain insight into its validity. To ensure content validity, we conducted a pilot survey and gathered feedback from one respiratory specialist, one obstetrics and gynecology specialist, two LAM patients, and three healthy participants regarding the questionnaire’s content, form, and structure. This study was approved by the Medical Ethics Committee of the First Affiliated Hospital of Guangzhou Medical University (Approval No. ES-2023-K007-01). All participants provided informed consent.

### Statistical analysis

R statistical software (version 4.1.2) and SPSS 26.0 software (SPSS, Chicago, IL, USA) were used for statistical analysis. Continuous variables that conformed to the normal distribution were expressed with mean (standard deviation), whereas variables that did not conform to the normal distribution were expressed with median (interquartile range). Multiple logistic regression was utilized to analyze how multiple factors affected adverse reactions. The occurrence of each adverse reaction was treated as the dependent variable, and whether the patient was a LAM patient was the independent variable. Various logistic regression models were developed to examine the impact of multiple factors on COVID-19 symptoms. Stepwise regression methods were adopted to control for potential confounding variables. A p value of < 0.05 indicated statistical significance.

## Results

### Vaccination against COVID-19 in the LAM population

This study included 324 participants: 143 healthy controls and 181 LAM patients. The mean age of the participants was 42.9 ± 9.51 years. Detailed information on the questionnaire can be found in Additional file 1, 2. In total, 67.9% of participants received three doses of the COVID-19 vaccine, and all healthy controls received inactivated vaccines. The vaccination rate among LAM patients was 77.34%. Out of these, 16 (8.84%) LAM patients received a vaccine other than an inactive vaccine. In addition, 100% of healthy controls had been infected with COVID-19, whereas only 72.4% of LAM patients had been infected. Basic characteristics of participants can be found in Additional file 3, Additional Table 1. Of the 181 patients with LAM, only 88 (48.6%) had oxygen saturation in the normal range. Besides, 149 (82.3%) regularly took mTOR inhibitors, of whom 67 (37.0%) used 0.1-1.0 mg per day, 15 (8.29%) used 1.5-2.0 mg per day, and 67 (37.0%) had an unknown dose. Additional file 3, Additional Table 2 summarizes the Clinical Characteristics of LAM Patients, which indirectly reflects the severity of LAM patients. After adjusting for confounders, we compared COVID-19 symptoms in LAM patients with different grades of daily oxygen saturation, and the results are in Additional file 3, Additional Table 3. Compared to LAM patients with 95–100% daily oxygen saturation, those with oxygen saturation below 90% were more likely to develop dyspnea [odds ratio (OR): 9.45, 95% confidence interval (CI): (1.96, 56.39)], whereas patients with oxygen saturation in 90–95% were less likely to develop hoarse throat [odds ratio (OR): 0.3, 95% confidence interval (CI): (0.09, 0.84)]. Risk analysis of COVID-19 symptoms in vaccinated healthy controls (*n* = 143) versus LAM patients (*n* = 104) is shown in Additional file 4, Additional Fig. 1.

### Reasons for COVID-19 vaccine hesitancy in LAM patients

**Fig. 2:**
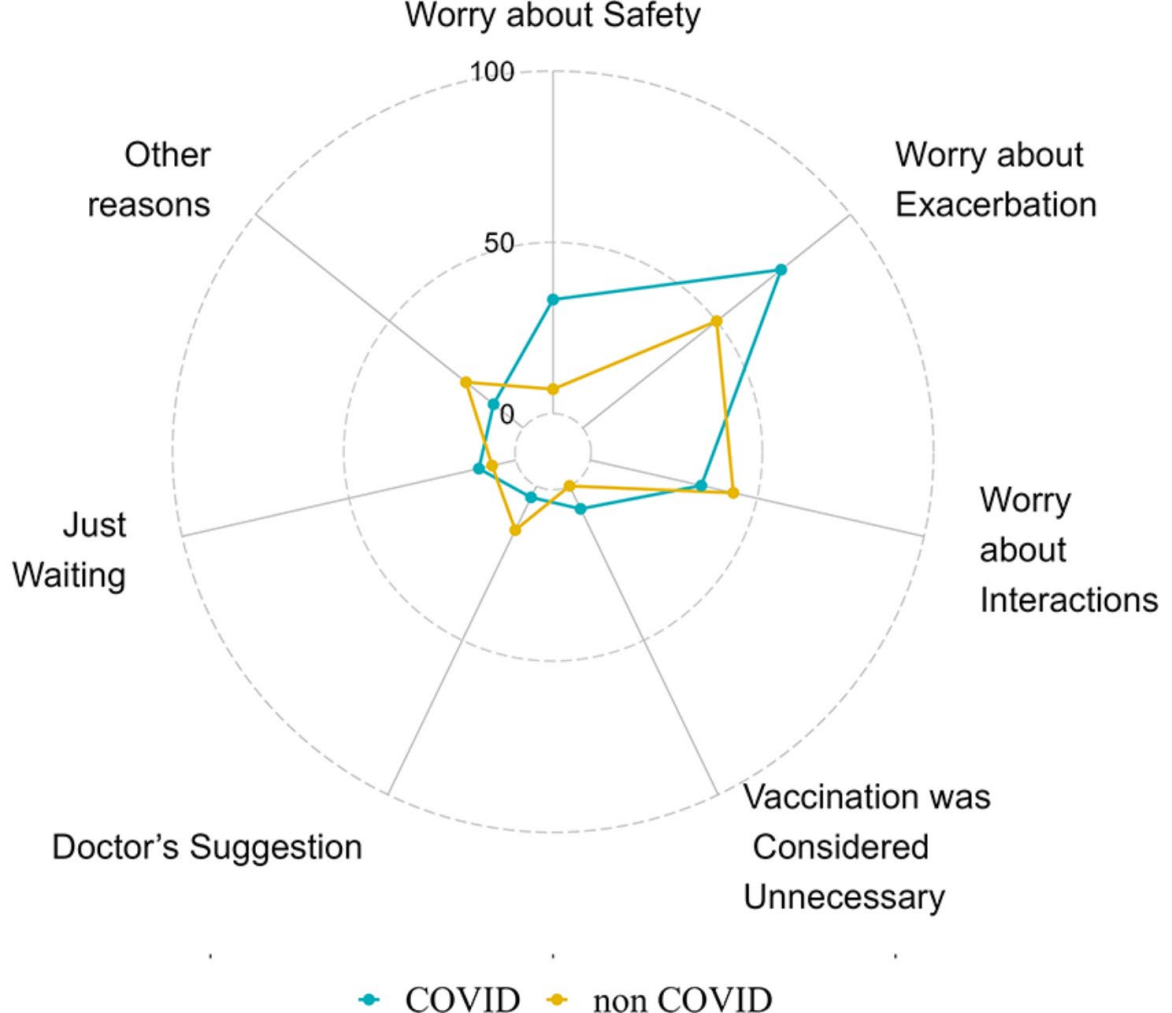
Reasons for not vaccinating lymphangioleiomyomatosis patients (*n* = 41)

A total of 41 LAM patients had not been vaccinated. The reasons for COVID-19 vaccine hesitancy in LAM patients were as follows: 27 concerned of disease aggravation (65.9%), 15 (36.6%) feared interactions between the vaccine and the medications used for LAM treatment, 10 (24.4%) feared LAM affecting the safety of the vaccine, four were skeptical about the safety of the COVID-19 vaccine (9.8%), three reported that their physicians did not recommend vaccination for them (7.3%), two thought that vaccination was not necessary (4.9%), and six chose not to vaccinate for other reasons (14.6%). Other reasons included “recent surgery,” “poor personal health,” “suffering from respiratory failure,” “preparing for pregnancy,” and “chronic eczema,” among other medical reasons (Fig. 2). More specific reasons for LAM patients’ hesitancy to vaccinate with COVID-19 can be found in Additional file 3, Additional Table 4.

### Safety of COVID-19 vaccines in LAM patients

In total, 42 individuals had adverse reactions after vaccination, of which 20 (14.0%) were from the healthy population and 22 (15.7%) from the LAM population. There were no significant differences between the two groups (*p* = 0.809). Detailed information about the occurrence of adverse reactions in the vaccination population is shown in Additional file 3, Additional Table 5.

**Fig. 3:**
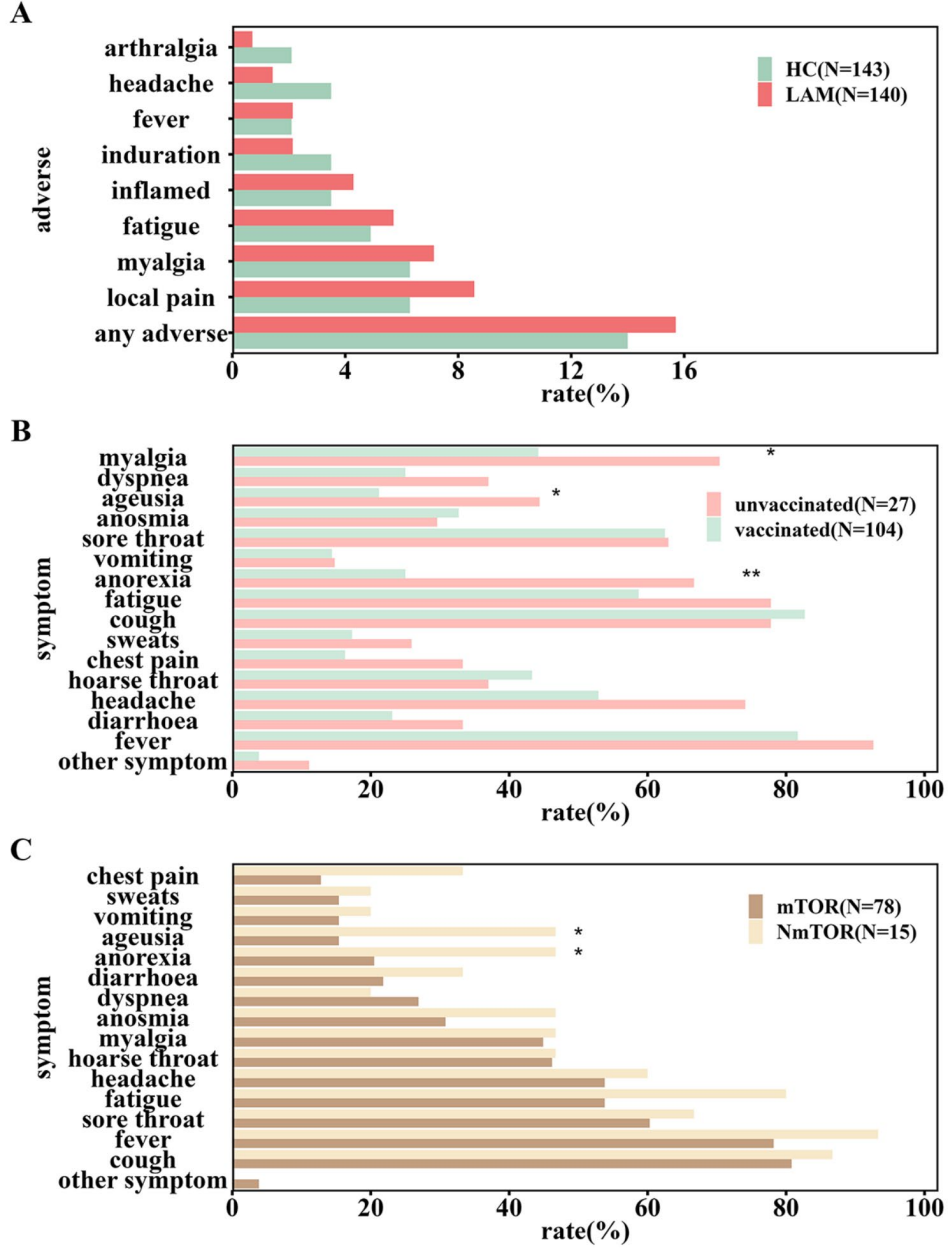
Histograms for each group analysis. (**A**) The rate of adverse reactions in vaccinated LAM patients versus healthy controls. (**B**) The rate of symptom onset in vaccinated versus unvaccinated LAM patients. (**C**) The rate of symptom onset in LAM patients taking versus not taking mTOR inhibitors regularly. HC, healthy controls; LAM, lymphangioleiomyomatosis; mTOR, LAM patients who have taken mammalian target of rapamycin (mTOR) inhibitors regularly; NmTOR, LAM patients who have not taken mTOR inhibitors regualrly; * *p* < 0.05, *** *p* < 0.001

The incidence of each adverse reaction was within 10% (Fig. 3A). The occurrence of adverse reactions after vaccination was similar in healthy controls and LAM patients. Among the adverse reactions, local pain was reported by the highest number of individuals (21 (7.42%) cases), followed by myalgia (19 (6.71%) cases). One case of grade 3 fever (above 39 degrees Celsius) was reported from the health control group and one case of grade 3 redness and swelling of vaccination site (diameter greater than 30 mm) was reported from the LAM group. All other adverse reactions were grade 1 or 2 and no hospitalization related to vaccine adverse reactions was reported within 6 months after vaccination. After adjusting for confounders such as age, BMI, underlying disease, number of vaccination doses, and hypertension, the occurrence of adverse reactions in LAM patients did not significantly differ from that in healthy controls [odds ratio (OR): 1.06, 95% confidence interval (CI): (0.53, 2.09), Fig. 3A, Additional file 5]. Subgroup analyses of LAM patients with and without adverse reactions to COVID-19 vaccination are shown in Additional file 3, Additional Table 6.

### The efficacy of COVID-19 vaccines in LAM patients

Of the 131 LAM patients infected with COVID-19, 27 were unvaccinated. The numbers of those with anorexia, ageusia, and myalgias were 44 (33.6%), 34 (26.0%), and 65 (49.6%), respectively. Additional file 3, Additional Table 7 provides basic information and symptoms for vaccinated and SARS-CoV-2 infected participants.

**Fig. 4:**
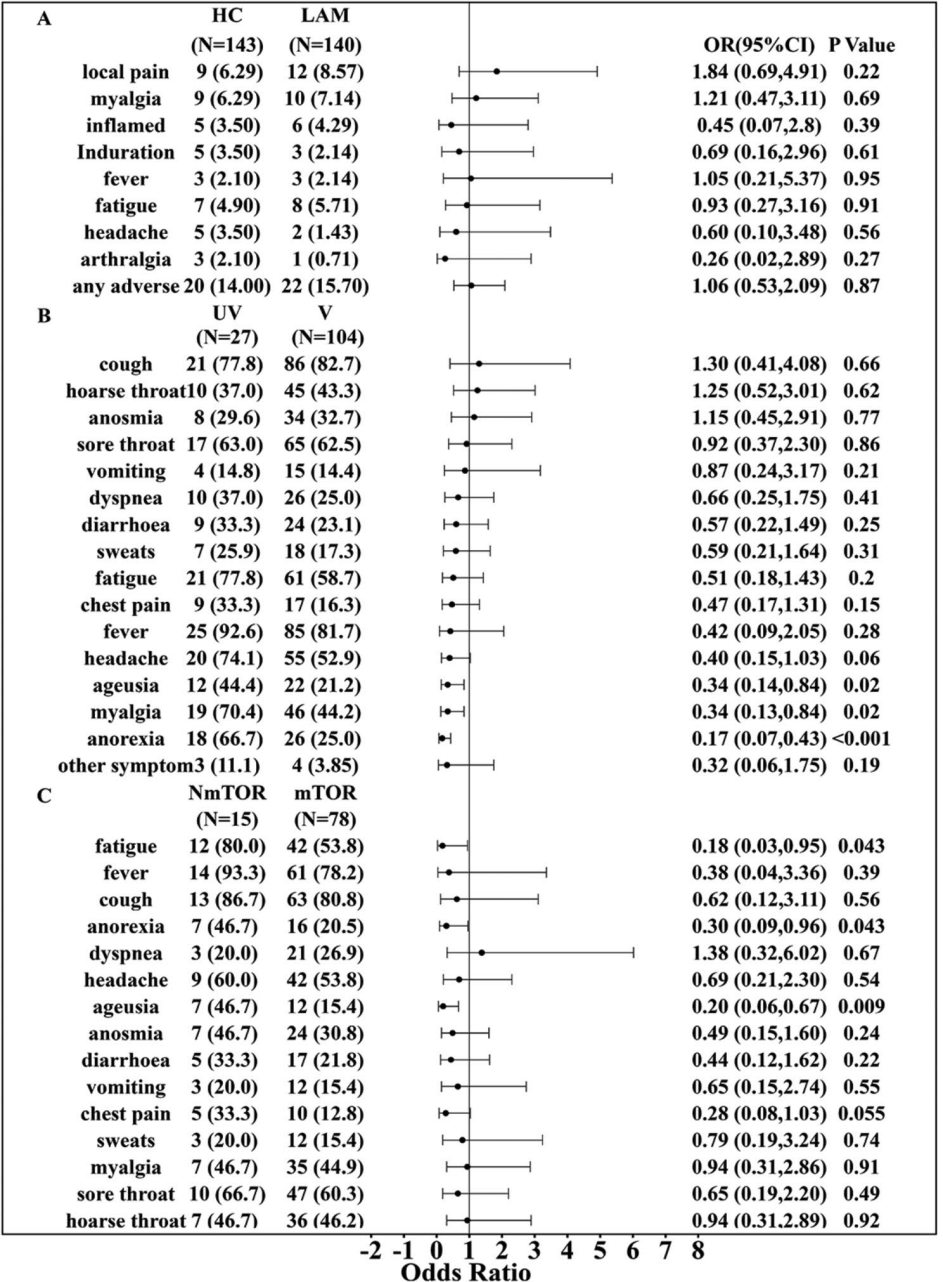
Forest plot of analyses by group. (**A**) Risk of symptom onset in vaccinated lymphangioleiomyomatosis (LAM) patients versus healthy controls (HC). (**B**) Risk of symptom exacerbation in vaccinated (V) versus unvaccinated (UV) LAM patients. (**C**) Risk of symptom onset in LAM patients regularly taking mammalian target of rapamycin (mTOR) inhibitors versus those not taking mTOR inhibitors regularly (NmTOR)

Both unvaccinated and vaccinated LAM patients infected with SARS-CoV-2 Omicron variant developed anorexia (*n* = 18, 66.7% and *n* = 26, 25.0%, respectively), ageusia (*n* = 12, 44.4% and *n* = 22, 21.2%, respectively), and myalgia (*n* = 19, 70.4% and *n* = 46, 44.2%, respectively). However, significant differences were observed between these two groups, with a corresponding OR of 0.17 (95% CI: 0.07–0.43) for anorexia and an OR of 0.34 (95% CI: 0.14–0.84) for ageusia (Figs. 3B and 4B). Adjusting for age, BMI and underlying disease condition revealed that vaccinated patients have a lower risk of experiencing anorexia [OR: 0.17, 95% CI: (0.07, 0.43)], myalgia [OR: 0.34, 95% CI: (0.13, 0.84)], and ageusia [OR: 0.34, 95% CI: (0.14, 0.84)]. The duration of symptoms did not significantly differ (Additional file 4, Additional Fig. 2). More information on basic characteristics and symptom occurrence in LAM patients can be found in Additional File 3, Additional Table 8. The basic characteristics of LAM patients did not significantly differ between the unvaccinated group and the vaccinated group. Furthermore, there was no significant difference in the rate of exacerbation in LAM-related symptoms such as fatigue, cough, dyspnea, chest tightness, chest pain, hemoptysis, pleural effusion and spontaneous pneumothorax between unvaccinated group and vaccinated group of patients during COVID-19, suggesting that the vaccine has a partially protective effect in LAM patients. The risks of symptom exacerbation in vaccinated versus unvaccinated LAM patients are shown in Additional file 4, Additional Fig. 4, Additional file 6 and Additional file 3, Additional Table 9. Details on the effect of regular mTOR inhibitors use on COVID-19 symptoms in LAM patients can be found in Additional file 3, Additional Table 10.

Unexpectedly, only the incidence of fatigue [OR: 0.26, 95% CI: (0.14,0.49)] differed in the incidence of COVID-19 symptoms between vaccinated LAM patients and healthy adults. More information about COVID-19 Symptoms in Vaccinated LAM Patients versus Healthy Adults is shown in Additional file 3, Additional Table 11. No hospitalization for COVID-19 was reported in the healthy control group. While two patients in the LAM group were hospitalized for the infection, one was vaccinated and developed a pneumothorax; the other was not vaccinated and developed respiratory failure, and both patients reported the use of supplemental oxygen.

### The effects of mTOR inhibitors on COVID-19 symptoms in LAM patients

Our survey revealed that the total number of LAM respondents who had received inactivated vaccines and were infected with SARS-CoV-2 Omicron variant was 93, of which 15 were not taking mTOR inhibitors. LAM patients who were taking mTOR inhibitors generally had a lower incidence of each symptom than those who had not taken the medication for a long time (Fig. 3C). Among them, LAM patients taking mTOR inhibitors regularly had a lower risk of fatigue [OR: 0.18, 95% CI: (0.03, 0.95)], anorexia [OR: 0.30, 95% CI: (0.09, 0.96)], and ageusia [OR: 0.20, 95% CI: (0.06, 0.67)]. Additional file 7 contains more information on the risk of COVID-19 symptom onset for mTOR medication versus no mTOR medication among LAM patients vaccinated with inactivated vaccine. The duration of symptoms did not significantly differ between patients taking and not taking mTOR inhibitors (Additional file 4, Additional Fig. 3).

## Discussion

To the best of our knowledge, no studies have reported on the safety and efficacy of COVID-19 vaccination in LAM patients during the COVID-19 Omicron pandemic. Our study has the largest sample size of LAM patients with COVID-19. We found that the COVID-19 vaccination rate in LAM patients was only 77.34%, indicating a need for improvement. Furthermore, in LAM patients, COVID-19 vaccines had no significant adverse effects (with an incidence of adverse reactions within the range of 10%) and decreased the occurrence of COVID-19 symptoms. In addition, the use of mTOR inhibitors was not associated with a higher risk of complications after COVID-19 in LAM patients.

Our study revealed that, as of January 2023, the estimated COVID-19 vaccination rate in LAM patients residing in China was approximately 77.34%. In China, the COVID-19 vaccine hesitancy rate is approximately 8% [19], with safety and efficacy being the most common reasons for hesitancy [20]. According to reports from July 23, 2022, the COVID-19 vaccination rate in mainland China was 89.7% [21], indicating that the vaccination rate among the Chinese LAM population is low. Other studies have reported a vaccine hesitancy rate of 17.8% in patients with chronic lung disease, which is comparable to our results [22]. Research into vaccine hesitancy among LAM patients found that the primary reasons were concerns about vaccine safety (65.9%) and uncertainty about the connection between sirolimus and the vaccine (36.6%). Thus, medical research should urgently investigate and report the safety of COVID-19 vaccination in the LAM population.

Our study found that LAM patients experienced adverse vaccine reactions at a rate of 15.7%, which is lower than that in previous studies and trials [23, 24]. The most frequently reported reactions were pain at the vaccination site (6.57%) and generalized myalgia (7.14%). No differences in vaccine side effects were observed compared to healthy controls, meaning that mTOR inhibitors do not increase the risk of adverse events in LAM patients with COVID-19 vaccination. Notably, no hospitalizations related to adverse reactions were reported within six months of vaccination. Thus, our study indicates the safety of COVID-19 vaccination in LAM patients taking sirolimus.

Chinese inactivated vaccines effectively reduce the rate of SARS-CoV-2 Omicron variant infection, hospitalization, severe disease, and mortality [25]. The inactivated vaccine also reduces infection and hospitalization rates in people with chronic lung disease [26, 27]. In our study, we observed the effect of COVID-19 vaccination on the incidence and duration of symptoms in LAM patients infected with the Omicron variant. COVID-19 vaccination reduced the risk of anorexia, myalgia, and ageusia in LAM patients. The incidence of other symptoms was also lower in the vaccinated compared to the unvaccinated group. In summary, the risk of COVID-19 vaccination with an inactivated virus vaccine in LAM patients is manageable and has some clinical benefits.

We compared the frequency and duration of symptoms between LAM patients taking mTOR inhibitors regularly and those who did not. Of note, the higher BMI in the group not taking mTOR inhibitors regularly may have led to more severe COVID-19 symptoms [28, 29]. Multivariate analysis indicated that patients in the group regularly using mTOR inhibitors exhibited a lower incidence of fatigue, ageusia, and anorexia. This underscores that mTOR inhibitors do not aggravate the symptoms of COVID-19 in LAM patients. On the contrary, mTOR inhibitors appear to mitigate the symptoms associated with COVID-19. Studies indicate that the phosphatidylinositol 3-kinase/protein kinase B/mTOR pathway is dysregulated in COVID-19 patients, affecting their immune response [30–32]. Inhibitors targeting the PI3K/Akt/mTOR pathway show anti-RNA viral effects, which could be therapeutic for COVID-19 [30–32]. Sirolimus, a well-known mTOR inhibitor, was approved by the U.S. Food and Drug Administration in 1999 as an anti-rejection medication after kidney transplantation. Sirolimus can inhibit several inflammatory factors, including the interleukins IL-2, IL-6, and IL-10. Thus, it contributes to regulating the synthesis of COVID-19 viral particles and cytokine storms, thereby helping control COVID-19 [14, 33]. Prior studies have reported that combining an mTOR inhibitor with hormones and antiviral drugs improves clinical outcomes, such as hypoxemia and multi-organ failure, in the treatment of Middle East respiratory syndrome and severe influenza [14, 33, 34]. Sirolimus mainly affects the host, and its effectiveness is less likely to be impacted by the high mutation rate of viral RNA. Therefore, it was deemed a promising treatment option for COVID-19 [33, 35]. Previous small-scale and international studies have reported that using mTOR inhibitors in LAM patients infected with SARS-CoV-2 Omicron variant does not result in adverse clinical outcomes [14, 36]. A Phase II double-blind clinical trial demonstrated that, compared to the placebo group, LAM patients hospitalized with COVID-19 who received sirolimus had a more rapid improvement in oxygenation and a shorter duration of hospital stay. However, the results were not statistically significant [37]. In terms of vaccination, sirolimus does not affect the anti-spike antibody levels in LAM patients after receiving the COVID-19 vaccine [38]. Nevertheless, these studies have limitations, such as a small sample size and/or the inability to eliminate confounding factors. Larger-scale research is required to evaluate the role of sirolimus in COVID-19 and vaccination. Our study results provide some evidence that sirolimus is safe for LAM patients post-COVID-19 and offers clinical justification for the aforementioned treatment.

Of course, this study has some limitations, including (1) the use of convenience sampling to include healthy controls, and the results are subject to selection bias (2) the information is based on patient reports, and the results are subject to recall bias, (3) the use of electronic questionnaires partially distributed online, and the responses may be affected by the participants’ conditions, (4) the lack of objective data such as pulmonary function parameters, radiological variables and other objective data, and (5) the LAM patients studied were all of yellow ethnicity from East Asia and lacked ethnic diversity. Therefore, multicenter international clinical studies are expected to be initiated in the future to provide further stronger evidence.

## Conclusion

This study is the first to investigate COVID-19 vaccine hesitancy, vaccine safety and efficacy, and the effect of mTOR inhibitors on COVID-19 symptoms in LAM patients during the Omicron pandemic. Our analysis of a large sample of LAM patients in China revealed that their vaccine hesitancy was due to concerns regarding vaccine safety and its interaction with mTOR inhibitors. In addition, our findings indicate the safety and efficacy of the COVID-19 vaccine in LAM patients, providing valuable evidence for clinical practice. Furthermore, we found that mTOR inhibitors did not exacerbate the symptoms of COVID-19 pneumonia in LAM patients.

### Electronic supplementary material

Below is the link to the electronic supplementary material.

Supplementary Material 1

Supplementary Material 2

Supplementary Material 3

Supplementary Material 4

Supplementary Material 5

Supplementary Material 6

Supplementary Material 7

## Data Availability

To safeguard the privacy of study participants, we cannot openly share the data. However, the datasets utilized or analyzed in this study can be obtained from the corresponding author upon reasonable request. Data are stored in controlled access data storage at the First Hospital of Guangzhou Medical University.

## Abbreviations

LAM: Lymphangioleiomyomatosis
S-LAM: Sporadic LAM
TSC-LAM: Tuberous sclerosis-associated pulmonary LAM
TSC: Tuberous sclerosis complex
mTOR: Mammalian target of rapamycin
SARS-CoV-2: Severe acute respiratory syndrome coronavirus 2
COVID-19: coronavirus disease 2019
BMI: body mass index
