# Supplementary material for: Safety and efficacy of COVID-19 vaccination in the Chinese population with pulmonary lymphangioleiomyomatosis: a single-center retrospective study
Source: Orphanet J Rare Dis. 2024 Jul 3;19:247. doi: 10.1186/s13023-024-03260-4 (PMC11220960; doi:10.1186/s13023-024-03260-4)
Supplement: Supplementary file 1 — Supplementary Material 1 [file 13023_2024_3260_MOESM1_ESM.docx]

# Additional file 1

# Questionnaire on the effect of COVID-19 vaccine and SARS-CoV-2 infection on LAM disease

Hello, you are cordially invited to participate in this study.

This study, conducted by Prof. Jie Liu's team at the First Hospital of Guangzhou Medical University and National Center for Respiratory Clinical Medicine, aims to collect information on COVID-19 vaccination and SARS-CoV-2 infection in patients with lymphangioleiomyomatosis (LAM) and to study its impact on LAM. This study will help explore how LAM patients can better cope with the COVID-19 epidemic and its long-term effects.

The information collected in this questionnaire is strictly confidential. You may also choose to fill out the survey anonymously by your Internet name, but please leave your personal contact information for future follow-up. We ask that you fill out the questionnaire honestly to the best of your ability. Thank you for participating in this study and you have the right to withdraw at any stage without any reason and without any loss of interest. Thank you very much for your participation!

I have read the above description of this study and have had the opportunity to discuss and ask questions about this study with the investigators, and the questions I have asked have been answered to my satisfaction.

I have been informed of the possible risks and benefits of participating in the study, I understand that participation in the study is voluntary, I acknowledge that I have had sufficient time to consider this, and I understand that:

I can ask the researcher for more information at any time.

I may withdraw from the study at any time without discrimination or reprisal, and my medical treatment and rights will not be affected

I am willing to receive follow-up visits after leaving my contact information.

I will be given a signed and dated copy of the informed consent form.

Finally, I agree to participate in this study and to cooperate as much as possible with the follow-up visits.

1. Will of the participants: (agree or disagree) Please press "Confirm and upload" at the bottom right after signing.

Part 1: Basic Imformations

1. Your gender

- Male
- Female

1. Your birth[fill-in-the-blanks]
2. Your height (cm) 【please answer the specific number, such as: 161】[fill-in-the-blanks]
3. Your weight(kg) 【answer with specific figures, please, e.g. 58】[fill-in-the-blanks]
4. Are you currently pregnant?

- Yes (e.g. 18 weeks pregnant) [fill-in-the-blanks]
- No

1. Have you ever smoked?

- Yes(e.g. For 5 years, 20 cigarettes per day) [fill-in-the-blanks]
- No

1. Have you quit smoking?

- Yes
- No

1. Other than lymphangioleiomyomatosis, do you have any other medical conditions or illnesses, such as hypertension, diabetes, coronary artery disease, renal malformation, chronic nephritis, or others?

- Yes [fill-in-the-blanks]
- No

Part 2: LAM condition

1. Do you have tuberous sclerosis other than lymphangioleiomyomatosis?

- Yes
- No
- Not sure

1. What was your age when you were diagnosed with lymphangioleiomyomatosis LAM?【Please answer with a specific number, e.g. 45 (years)】[fill-in-the-blanks]
2. What symptoms did you have when you were first diagnosed with lymphangioleiomyomatosis (LAM)? [Multiple choice].

- Lack of power
- Dyspnea
- Spontaneous pneumothorax or pulmonary atelectasis
- Pleural effusion (fluid around the lungs)
- Hemoptysis (blood in sputum)
- Asymptomatic (found unintentionally by examination for other reasons)
- Chest pain
- Cough
- Chest tightness
- Other symptoms[fill-in-the-blanks]

1. Your daily blood oxygen test value or the last blood oxygen measurement 【please enter a specific number, e.g. 96%】[fill-in-the-blanks]
2. Are you regularly using sirolimus or everolimus for the treatment of

lymphangioleiomyomatosis (LAM)

- Yes
- No

1. What is the dose you are using (several times a day, a few grams each time) [fill-in-the-blanks]
2. Are you taking any other medications on a long-term basis?

- Yes
- No

1. Please enter the name of the drug and the dose (drug, several times a day, a few milligrams each time) [fill-in-the-blanks]

Part 2: Vaccine related information（You can check the vaccination record of COVID-19 by clicking " COVID-19 Vaccine" at the bottom right corner of Yue Kang Code.）

1. Have you received the COVID vaccine?

- Yes
- No

1. If you have not received the COVID-19 vaccine, what is your reason for not getting vaccinated? [Multiple Choice]

- Concerned that LAM affects the safety of the vaccine
- Worried that the vaccine would aggravate LAM
- Worried about the interaction between vaccine and therapeutic drugs
- Don't think vaccination is necessary
- Doctor did not recommend vaccination for LAM patients
- Just waiting
- Other[fill-in-the-blanks]

1. What symptoms did you have before the COVID-19 vaccination? [Multiple Choice]

- Lack of power
- Dyspnea
- spontaneous pneumothorax or atelectasis
- pleural effusion (fluid around the lungs)
- hemoptysis (blood in sputum)
- asymptomatic (found unintentionally by examination for other reasons)
- chest pain
- cough
- chest tightness
- other symptoms (please specify) [fill-in-the-blank]
- No symptoms, first symptoms disappeared after treatment

1. Did you stop taking sirolimus before and after the COVID-19 vaccination?

- Yes
- No

1. Which dose of the COVID-19 vaccine have you received?

- First dose
- Second dose
- Third dose
- Fourth dose

1. What type of vaccine did you receive for your first dose of COVID-19?

- Inactivated vaccines
- recombinant protein vaccines
- adenovirus vector vaccines
- influenza virus vector vaccines

1. What type of vaccine did you receive for your second dose of COVID-19?

- Inactivated vaccines
- recombinant protein vaccines
- adenovirus vector vaccines
- influenza virus vector vaccines

1. What type of vaccine did you receive for your third dose of COVID-19?

- Inactivated vaccines
- recombinant protein vaccines
- adenovirus vector vaccines
- influenza virus vector vaccines

1. What type of vaccine did you receive for your forth dose of COVID-19?

- Inactivated vaccines
- recombinant protein vaccines
- adenovirus vector vaccines
- influenza virus vector vaccines

1. Did you have an adverse reaction, within seven days of your COVID-19 vaccination?

- Yes
- No

1. What adverse reactions have you experienced? [ Multiple Choice Questions]

- Redness and swelling of the inoculation site
- Hard nodules at the inoculation site
- Pain at the inoculation site
- Fever
- Fatigue
- Nausea, vomiting
- Headache
- Muscle aches and pains
- Diarrhea
- Joint pain
- Other[fill-in-the-blanks]

1. What is the redness and swelling of your vaccination site?

- Diameter less than 15mm
- Between 15mm and 30mm in diameter
- Diameter greater than 30mm
- Gangrene (localized darkening, decay and necrosis) or exfoliative dermatitis (diffuse erythema, swelling and flaking of the skin over the whole body or over a large area)

1. What is the condition of the hard knot at your vaccination site?

- Diameter less than 15mm
- Between 15mm and 30mm in diameter
- Diameter greater than 30mm
- Gangrene (localized darkening, decay and necrosis) or exfoliative dermatitis (diffuse erythema, swelling and flaking of the skin over the whole body or over a large area)

1. What is the pain at your vaccination site?

- Does not affect normal life
- Interferes with activity or repeated use of non-narcotic pain medication
- Interferes with daily life or repeated use of narcotic painkillers (e.g. morphine)
- Emergency medical treatment or hospitalization

1. What is your fever? (Axillary temperature)

- 37.1 degrees Celsius to 37.5 degrees Celsius
- 37.6 degrees Celsius to 39 degrees Celsius
- Above 39 degrees Celsius

1. What is your fatigue?

- Normal activity diminished for <48 hours, does not affect activity
- 20%-50% reduction in normal activity >48 hours, slightly affecting activity
- Normal activity is reduced >50%, seriously affects daily activities, unable to work
- Unable to take care of yourself, emergency treatment or hospitalization

1. What is your nausea and vomiting?

- 1~2 times/24 hours, intake is basically normal and does not affect activity
- 2 to 5 times/24 hours, significantly reduced intake, or limited activity
- >6 times in 24 hours, no significant intake, need for IV fluids
- Need for hospitalization or other means of nutrition due to hypotensive shock

1. What is the condition of your headache?

- Does not interfere with activity and does not require treatment.
- Occurs once in a short period of time, slightly interferes with activity, requires treatment (multiple use of non-narcotic pain medications such as ibuprofen, acetaminophen).
- Severely interferes with daily activities, responds to initial narcotic treatment
- Intractable, repeated narcotic treatment. Emergency or hospitalization.

1. What is your muscle pain?

- Does not affect activity, no treatment needed
- Tenderness in non-injection site muscles, slightly affecting daily activities
- Severe muscle tenderness, severely affecting daily activities
- Significantly symptomatic, muscle necrosis, emergency or hospitalization

1. What is the condition of your diarrhea?

- Once in a short period of time, 2-3 loose stools/day, or mild diarrhea lasting less than 1 week
- Persistent diarrhea, 4-5 times/day, or diarrhea >1 week
- >6 watery stools/day, or bloody diarrhea, upright hypotension, electrolyte imbalance, requiring intravenous fluids >2L
- Hypotensive shock requiring hospitalization

1. What is your joint pain?

- Does not affect movement
- Slightly affects daily activities
- Severely affects daily activities
- Significantly affects your daily activities, emergency or hospitalization

1. Have you had any of the following changes in your symptoms within two weeks of your vaccination?[ Multiple Choice Questions]

- Increased fatigue
- Increased dyspnea
- Development of spontaneous pneumothorax or pulmonary atelectasis
- Increased pleural effusion (peri-pulmonary effusion)
- Increased hemoptysis (blood in sputum)
- Increased chest pain
- worsening of cough
- Increased chest tightness
- Other symptoms worsened[fill-in-the-blanks]
- No significant change

1. Have you been hospitalized within 6 months of vaccination?

- Yes
- No

1. Reasons for your admission[ Multiple Choice Questions]

- Lack of power
- Difficulty breathing
- Spontaneous pneumothorax or pulmonary atelectasis
- Pleural effusion (fluid around the lungs)
- Hemoptysis (blood in sputum)
- Chest pain
- Cough
- Chest tightness
- Other symptoms[fill-in-the-blanks]
- Not related to LAM[fill-in-the-blanks]

Part 3 COVID-19 related information

1. Are you infected with the COVID-19?

- Yes
- No
- Not sure

1. How do you know you have a SARS-CoV-2 infection?

- Positive nucleic acid test
- Positive antigen test
- Symptoms of suspected SARS-CoV-2 infection
- Other[fill-in-the-blanks]

1. What symptoms did you have before you contracted COVID-19? [Multiple Choice]

- Lack of power
- Difficulty breathing
- Spontaneous pneumothorax or pulmonary atelectasis
- Pleural effusion (fluid around the lungs)
- Hemoptysis (blood in sputum)
- Chest pain
- Cough
- Chest tightness
- Other symptoms[fill-in-the-blanks]
- No symptoms (disappearance of first symptoms after treatment)

1. What are the changes in your symptoms after your SARS-CoV-2 infection [multiple choice].

- Worse fatigue
- Worse dyspnea
- Development of spontaneous pneumothorax (pulmonary atelectasis)
- Increased pleural effusion (peripulmonary effusion)
- Worsening hemoptysis (blood in sputum)
- Worsening of chest pain
- Worsening of cough
- Worsening of chest tightness
- Aggravation of other symptoms[fill-in-the-blanks]
- No significant change

1. After your SARS-CoV-2 infection, what was your sirolimus dosing status?

- Maintain the same dose and frequency
- Reduce the dose and frequency;
- Stop taking

1. After the onset of a COVID-19, which of the following symptoms do you experience[Multiple Choice]

- lack of power
- Fever
- Cough
- Anorexia
- Difficulty breathing
- Headache
- Loss of taste
- Abnormal sense of smell
- Diarrhea
- Vomiting
- Chest pain
- Night sweats
- Muscle pain
- Sore throat
- Hoarse throat
- Other[fill-in-the-blanks]
- No significant discomfort (unintentionally found during testing for other reasons)

1. What is the highest degree when you have a fever? 【Please enter a specific number, e.g. 39.5℃】[fill-in-the-blanks]
2. How many days have you had fever over 38℃?【Please enter the specific number, such as 1 day, if the fever temperature is below 38℃, please fill in 0】[fill-in-the-blanks]
3. Have you already taken the COVID-19 drug paxlovid

- Yes
- No

1. Have you been hospitalized for a SARS-CoV-2 infection?

- Yes
- no

1. The reason you were admitted to the hospital after your SARS-CoV-2 infection was

fatigue [ Multiple Choice Questions]

- Lack of power
- Difficulty breathing
- Spontaneous pneumothorax or pulmonary atelectasis
- Pleural effusion (fluid around the lungs) Hemoptysis (blood in the sputum)
- Chest pain
- Cough
- Chest tightness
- Other symptoms [fill-in-the-blanks]
- Not related to LAM[fill-in-the-blanks]

1. What conditions occurred during your hospitalization [multiple choice]

- pneumothorax
- pulmonary embolism
- oxygen therapy (please specify if it was the first time you received oxygen) [fill-in-the-blanks]
- mechanical ventilation
- other[fill-in-the-blanks]

Part 5: Follow-up visits

1. If we have questions about the answer, how would you like us to contact you?

Mobile; Email, WeChat; Others[fill-in-the-blanks] [ Multiple Choice Questions]

1. Your phone number[fill-in-the-blanks]
2. Your email address[fill-in-the-blanks]
3. Your micro signal[fill-in-the-blanks]
4. Your name[fill-in-the-blanks]
