# Supplementary material for: Safety and efficacy of COVID-19 vaccination in the Chinese population with pulmonary lymphangioleiomyomatosis: a single-center retrospective study
Source: Orphanet J Rare Dis. 2024 Jul 3;19:247. doi: 10.1186/s13023-024-03260-4 (PMC11220960; doi:10.1186/s13023-024-03260-4)
Supplement: Supplementary file 2 — Supplementary Material 2 [file 13023_2024_3260_MOESM2_ESM.docx]

**Additional file 2**

**Questionnaire on COVID-19 vaccination and SARS-CoV-2 infection in women over 16 years of age**

Hello, you are cordially invited to participate in this study.

This study, conducted by Professor Liu Jie's team at the First Hospital of Guangzhou Medical University and the National Center for Respiratory Clinical Medicine, aims to cell phone information on COVID-19 vaccination and SARS-CoV-2 infection in healthy people and patients with lymphangioleiomyomatosis (LAM), and to help LAM patients better cope with the COVID-19 epidemic and its long-term effects through comparative analysis of data with healthy people

This questionnaire is intended for healthy people without LAM

The information collected in this survey is strictly confidential. You may also choose to

to fill out the survey anonymously, but please leave your personal contact information for

for our future follow-up. We ask that you fill out the survey truthfully. Thank you for participating in this study, and you have the right to withdraw at any stage without reason and without any loss of interest. and there will be no loss of interest. Thank you very much for your participation!

I have been informed of the possible risks and benefits of participating in the study, I understand that participation in the study is voluntary, I acknowledge that I have had sufficient time to consider this, and I understand that:

- I can ask the researcher for more information at any time.
- I may withdraw from the study at any time without discrimination or reprisal, and my medical treatment and rights will not be affected
- I am willing to receive follow-up visits after leaving my contact information.

I will be given a signed and dated copy of the informed consent form.

Finally, I agree to participate in this study and to cooperate as much as possible with the follow-up visits.

1. Will of the participants: (agree or disagree) Please press "Confirm and upload" at the bottom right after signing.

Part 1: Basic Imformations

2. Your gender

- Male
- Female

3. Your birth[fill-in-the-blanks]

4. Your orgin[fill-in-the-blanks]

5. Your Height(cm)【answer with specific figures, please, e.g. 161】[fill-in-the-blanks]

6. Your weight(kg) 【answer with specific figures, please, e.g. 58】[fill-in-the-blanks]

7. Are you currently pregnant?

- Yes (e.g. 18 weeks pregnant) [fill-in-the-blanks]
- No

8. Have you ever smoked?

- Yes(e.g. For 5 years, 20 cigarettes per day) [fill-in-the-blanks]
- No

9. Have you quit smoking?

- Yes
- No

10. Do you suffer from diseases? 【such as hypertension, diabetes, coronary heart disease, chronic obstructive pulmonary disease, chronic nephritis, malignancy, immune function...】

- Yes[fill-in-the-blanks]
- No

11. Have you ever received an organ transplant?

- Yes【e.g. right lung transplant】[fill-in-the-blanks]
- No

Part 2: Vaccine related information（You can check the vaccination record of COVID-19 by clicking " COVID-19 Vaccine" at the bottom right corner of Yue Kang Code.）

12. Have you received the COVID vaccine?

- Yes
- no

13. Did you have any of the following symptoms prior to the COVID-19 vaccination?[ Multiple Choice Questions]

- Lack of power
- Cough
- chest tightness
- Hemoptysis (blood in sputum)
- Chest pain
- Difficulty in breathing
- Pleural effusion (fluid around the lungs)
- Spontaneous pneumothorax (atelectasis of the lung)
- Other[fill-in-the-blanks]
- None

14. Which dose of the COVID-19 vaccine have you received?

- First vaccination
- Second vaccination
- Third vaccination
- Fourth vaccination

15. What type of vaccine did you receive for your first dose of COVID-19?

- Inactivated vaccines
- recombinant protein vaccines
- adenovirus vector vaccines
- influenza virus vector vaccines

16. What type of vaccine did you receive for your second dose of COVID-19?

- Inactivated vaccines
- recombinant protein vaccines
- adenovirus vector vaccines
- influenza virus vector vaccines

17. What type of vaccine did you receive for your third dose of COVID-19?

- Inactivated vaccines
- recombinant protein vaccines
- adenovirus vector vaccines
- influenza virus vector vaccines

18. What type of vaccine did you receive for your forth dose of COVID-19?

- Inactivated vaccines
- recombinant protein vaccines
- adenovirus vector vaccines
- influenza virus vector vaccines

19. Did you have an adverse reaction, within seven days of your COVID-19 vaccination

- Yes
- No

20. What adverse reactions have you experienced? [ Multiple Choice Questions]

- Redness and swelling of the inoculation site
- Hard nodules at the inoculation site
- Pain at the inoculation site
- Fever
- Fatigue
- Nausea, vomiting
- Headache
- Muscle aches and pains
- Diarrhea
- Joint pain
- Other[fill-in-the-blanks]

21. What is the redness and swelling of your vaccination site?

- Diameter less than 15mm
- Between 15mm and 30mm in diameter
- Diameter greater than 30mm
- Gangrene (localized darkening, decay and necrosis) or exfoliative dermatitis (diffuse erythema, swelling and flaking of the skin over the whole body or over a large area)

22. What is the condition of the hard knot at your vaccination site?

- Diameter less than 15mm
- Between 15mm and 30mm in diameter
- Diameter greater than 30mm
- Gangrene (localized darkening, decay and necrosis) or exfoliative dermatitis (diffuse erythema, swelling and flaking of the skin over the whole body or over a large area)

23. What is the pain at your vaccination site?

- Does not affect normal life
- Interferes with activity or repeated use of non-narcotic pain medication
- Interferes with daily life or repeated use of narcotic painkillers (e.g. morphine)
- Emergency medical treatment or hospitalization

24. What is your fever? (Axillary temperature)

- 37.1 degrees Celsius to 37.5 degrees Celsius
- 37.6 degrees Celsius to 39 degrees Celsius
- Above 39 degrees Celsius

25.What is your fatigue?

- Normal activity diminished for <48 hours, does not affect activity
- 20%-50% reduction in normal activity >48 hours, slightly affecting activity
- Normal activity is reduced >50%, seriously affects daily activities, unable to work
- Unable to take care of yourself, emergency treatment or hospitalization

26.What is your nausea and vomiting?

- 1~2 times/24 hours, intake is basically normal and does not affect activity
- 2 to 5 times/24 hours, significantly reduced intake, or limited activity
- >6 times in 24 hours, no significant intake, need for IV fluids
- Need for hospitalization or other means of nutrition due to hypotensive shock

1. What is the condition of your headache?

- Does not interfere with activity and does not require treatment.
- Occurs once in a short period of time, slightly interferes with activity, requires treatment (multiple use of non-narcotic pain medications such as ibuprofen, acetaminophen).
- Severely interferes with daily activities, responds to initial narcotic treatment
- Intractable, repeated narcotic treatment. Emergency or hospitalization.

28. What is your muscle pain?

- Does not affect activity, no treatment needed
- Tenderness in non-injection site muscles, slightly affecting daily activities
- Severe muscle tenderness, severely affecting daily activities
- Significantly symptomatic, muscle necrosis, emergency or hospitalization

29. What is the condition of your diarrhea?

- Once in a short period of time, 2-3 loose stools/day, or mild diarrhea lasting less than 1 week
- Persistent diarrhea, 4-5 times/day, or diarrhea >1 week
- >6 watery stools/day, or bloody diarrhea, upright hypotension, electrolyte imbalance, requiring intravenous fluids >2L
- Hypotensive shock requiring hospitalization

30. What is your joint pain?

- Does not affect movement
- Slightly affects daily activities
- Severely affects daily activities
- Significantly affects your daily activities, emergency or hospitalization

31. Have you had any of the following changes in your symptoms within two weeks of your vaccination?[ Multiple Choice Questions]

- Increased fatigue
- Increased dyspnea
- Development of spontaneous pneumothorax or pulmonary atelectasis
- Increased pleural effusion (peri-pulmonary effusion)
- Increased hemoptysis (blood in sputum)
- Increased chest pain
- worsening of cough
- Increased chest tightness
- Other symptoms worsened[fill-in-the-blanks]
- No significant change

32. Have you been hospitalized within 6 months of vaccination?

- Yes
- No

33. Reasons for your admission[ Multiple Choice Questions]

- lack of power
- Difficulty breathing
- Spontaneous pneumothorax or pulmonary atelectasis
- Pleural effusion (fluid around the lungs)
- Hemoptysis (blood in sputum)
- Chest pain
- Cough
- Chest tightness
- Other symptoms[fill-in-the-blanks]

Part 3 COVID-19 related information

34. Are you infected with the COVID-19?

- Yes
- No
- Not sure

35. Reasons why you suspect you have a SARS-CoV-2 infection

- The appearance of COVID-19s with similar symptoms
- Other[fill-in-the-blanks]

36. How do you know you have a SARS-CoV-2 infection?

- Positive nucleic acid test
- Positive antigen test
- Symptoms of suspected SARS-CoV-2 infection
- Other[fill-in-the-blanks]

37. When did you get the COVID-19?

- Within a week
- Within two weeks
- Within 3 weeks
- Within 4 weeks
- Within 5 weeks
- Within 6 weeks
- Within 2 months
- Other[fill-in-the-blanks]

38. Which of the following symptoms occur in your first and second week after the onset of a COVID-19[ Multiple Choice Questions]

- lack of power
- Fever
- Cough
- Anorexia
- Difficulty breathing
- Headache
- Loss of taste
- Abnormal sense of smell
- Diarrhea
- Vomiting
- Chest pain
- Night sweats
- Muscle pain
- Sore throat
- Hoarse throat
- Other[fill-in-the-blanks]
- No significant discomfort (unintentionally found during testing for other reasons)

39. Which of the following symptoms occur in your 3rd and 4th week after the onset of a COVID-19[ Multiple Choice Questions]

- lack of power
- Fever
- Cough
- Anorexia
- Difficulty breathing
- Headache
- Loss of taste
- Abnormal sense of smell
- Diarrhea
- Vomiting
- Chest pain
- Night sweats
- Muscle pain
- Sore throat
- Hoarse throat
- Other[fill-in-the-blanks]
- No significant discomfort (unintentionally found during testing for other reasons)

40. Which of the following symptoms occur during your 5th and 6th week after the onset of a COVID-19[ Multiple Choice Questions]

- lack of power
- Fever
- Cough
- Anorexia
- Difficulty breathing
- Headache
- Loss of taste
- Abnormal sense of smell
- Diarrhea
- Vomiting
- Chest pain
- Night sweats
- Muscle pain
- Sore throat
- Hoarse throat
- Other[fill-in-the-blanks]
- No significant discomfort (unintentionally found during testing for other reasons)

41. What is the highest degree when you have a fever? 【Please enter a specific number, e.g. 39.5℃】[fill-in-the-blanks]

42. How many days have you had fever over 38℃?【Please enter the specific number, such as 1 day, if the fever temperature is below 38℃, please fill in 0】

[fill-in-the-blanks]

43. Have you already taken the COVID-19 drug paxlovid

- Yes
- No

44. Your visit to the doctor after the SARS-CoV-2 infection was

- Did not seek medical attention
- emergency
- Outpatient
- Online consultation

45. Have you been hospitalized for a SARS-CoV-2 infection?

- Yes
- no

46. The reason you were admitted to the hospital after your SARS-CoV-2 infection was

fatigue [ Multiple Choice Questions]

- Lack of power
- Difficulty breathing
- Spontaneous pneumothorax or pulmonary atelectasis
- Pleural effusion (fluid around the lungs) Hemoptysis (blood in the sputum)
- Chest pain
- Cough
- Chest tightness
- Other symptoms [fill-in-the-blanks]

47. Your oxygen intake status during your hospital stay was[ Multiple Choice Questions]

- No oxygen
- on oxygen
- Mechanical Ventilation
- Other[fill-in-the-blanks]

48. What happened to you during the COVID-19[ Multiple Choice Questions]

- Pneumonia
- Pulmonary embolism
- Respiratory failure
- Shock
- Pneumothorax
- Other[fill-in-the-blanks]
- None

49. Have all the symptoms you experienced after the SARS-CoV-2 infection disappeared so far? (such as fatigue, fever, cough, anorexia, dyspnea...)

- Yes
- no

50. As of now, how many days have you been experiencing symptoms after the SARS-CoV-2 infection? (Please enter the exact number of days, e.g. 18) [fill-in-the-blanks]

Part 4: Follow-up visits

51. If we have questions about the answer, how would you like us to contact you?

Mobile; Email, WeChat; Others[fill-in-the-blanks] [ Multiple Choice Questions]

52. Your phone number[fill-in-the-blanks]

53. Your email address[fill-in-the-blanks]

54.Your micro signal[fill-in-the-blanks]

55.Your name[fill-in-the-blanks]
