# Supplementary material for: Safety and efficacy of COVID-19 vaccination in the Chinese population with pulmonary lymphangioleiomyomatosis: a single-center retrospective study
Source: Orphanet J Rare Dis. 2024 Jul 3;19:247. doi: 10.1186/s13023-024-03260-4 (PMC11220960; doi:10.1186/s13023-024-03260-4)
Supplement: Supplementary file 3 — Supplementary Material 3 [file 13023_2024_3260_MOESM3_ESM.docx]

**Additional file 3:**

**Additional Table 1: Basic characteristics of participants**

|  | **[ALL]**  ***N=324*** | **HC**  ***N=143*** | **LAM**  ***N=181*** | **p.overall** |
| --- | --- | --- | --- | --- |
| Agee mean(SD) | 42.9 (9.51) | 42.8 (9.08) | 42.9 (9.87) | 0.946 |
| Age stratification: |  |  |  | 0.160 |
| 19~40 | 117 (36.10%) | 44 (30.80%) | 73 (40.30%) |  |
| 40~60 | 194 (59.90%) | 94 (65.70%) | 100 (55.20%) |  |
| >=60 | 13 (4.01%) | 5 (3.50%) | 8 (4.42%) |  |
| BMI | 21.5 (3.29) | 22.7 (2.59) | 20.6 (3.50) | <0.001 |
| BMI stratification: |  |  |  | <0.001 |
| <18.5 | 39 (12.00%) | 2 (1.40%) | 37 (20.40%) |  |
| 18.5~23.9 | 217 (67.00%) | 96 (67.10%) | 121 (66.90%) |  |
| >=23.9 | 66 (20.40%) | 45 (31.50%) | 21 (11.60%) |  |
| 'Missing' | 2 (0.62%) | 0 (0.00%) | 2 (1.10%) |  |
| Smoking: |  |  |  | 0.139 |
| No | 317 (97.80%) | 142 (99.30%) | 175 (96.70%) |  |
| Yes | 7 (2.16%) | 1 (0.70%) | 6 (3.31%) |  |
| Underlying disease: |  |  |  | 0.003 |
| No | 277 (85.50%) | 132 (92.30%) | 145 (80.10%) |  |
| Yes | 47 (14.50%) | 11 (7.69%) | 36 (19.90%) |  |
| Hypertension: |  |  |  | 0.025 |
| No | 291 (89.80%) | 135 (94.40%) | 156 (86.20%) |  |
| Yes | 33 (10.20%) | 8 (5.59%) | 25 (13.80%) |  |
| Coronary heart disease: |  |  |  | 1.000 |
| No | 321 (99.10%) | 142 (99.30%) | 179 (98.90%) |  |
| Yes | 3 (0.93%) | 1 (0.70%) | 2 (1.10%) |  |
| Diabetes: |  |  |  | 0.133 |
| No | 320 (98.80%) | 143 (100%) | 177 (97.80%) |  |
| Yes | 4 (1.23%) | 0 (0.00%) | 4 (2.21%) |  |
| Tumor: |  |  |  | 0.471 |
| No | 317 (97.80%) | 141 (98.60%) | 176 (97.20%) |  |
| Yes | 7 (2.16%) | 2 (1.40%) | 5 (2.76%) |  |
| Renal insufficiency: |  |  |  | 1.000 |
| No | 323 (99.70%) | 143 (100%) | 180 (99.40%) |  |
| Yes | 1 (0.31%) | 0 (0.00%) | 1 (0.55%) |  |
| Autoimmune disease: |  |  |  | 0.133 |
| No | 320 (98.80%) | 143 (100%) | 177 (97.80%) |  |
| Yes | 4 (1.23%) | 0 (0.00%) | 4 (2.21%) |  |
| Tuberous sclerosis: |  |  |  | <0.001 |
| S-LAM | 130 (40.10%) | 0 (0.00%) | 130 (71.80%) |  |
| TSC-LAM | 19 (5.86%) | 0 (0.00%) | 19 (10.50%) |  |
| No | 175 (54.00%) | 143 (100%) | 32 (17.70%) |  |
| Sirolimus or everolimus: |  |  |  | <0.001 |
| No | 32 (9.88%) | 0 (0.00%) | 32 (17.70%) |  |
| Yes | 149 (46.00%) | 0 (0.00%) | 149 (82.30%) |  |
| 'Missing' | 143 (44.10%) | 143 (100%) | 0 (0.00%) |  |
| Vaccine dose: |  |  |  | <0.001 |
| 1dose | 8 (2.47%) | 3 (2.10%) | 5 (2.76%) |  |
| 2dose | 44 (13.60%) | 13 (9.09%) | 31 (17.10%) |  |
| 3dose | 220 (67.90%) | 118 (82.50%) | 102 (56.40%) |  |
| 4dose | 11 (3.40%) | 9 (6.29%) | 2 (1.10%) |  |
| 'Missing' | 41 (12.70%) | 0 (0.00%) | 41 (22.70%) |  |
| Vaccine Type: |  |  |  | <0.001 |
| Unvaccinated | 41 (12.70%) | 0 (0.00%) | 41 (22.70%) |  |
| Inactivated Vaccine | 267 (82.40%) | 143 (100%) | 124 (68.50%) |  |
| Other vaccines | 16 (4.94%) | 0 (0.00%) | 16 (8.84%) |  |
| COVID-19: |  |  |  | <0.001 |
| No | 50 (15.40%) | 0 (0.00%) | 50 (27.60%) |  |
| Yes | 274 (84.60%) | 143 (100%) | 131 (72.40%) |  |

**Additional Table 2: Clinical Characteristics of LAM Patients**

|  | **[ALL]** | **S-LAM** | **TSC-LAM** | **No** | **p.overall** |
| --- | --- | --- | --- | --- | --- |
|  | ***N=181*** | ***N=130*** | ***N=19*** | ***N=32*** |  |
| age | 42.9 (9.87) | 43.4 (9.31) | 39.4 (11.9) | 43.2 (10.6) | 0.253 |
| Daily_oxygen_saturation: |  |  |  |  | 0.026 |
| 96-100 | 88 (48.60%) | 70 (53.80%) | 6 (31.60%) | 12 (37.50%) |  |
| 90-95 | 44 (24.30%) | 31 (23.80%) | 5 (26.30%) | 8 (25.00%) |  |
| <=90 | 16 (8.84%) | 13 (10.00%) | 0 (0.00%) | 3 (9.38%) |  |
| 'Missing' | 33 (18.20%) | 16 (12.30%) | 8 (42.10%) | 9 (28.10%) |  |
| Dose_of_mTOR_cat: |  |  |  |  | 0.077 |
| 0 | 32 (17.70%) | 23 (17.70%) | 2 (10.50%) | 7 (21.90%) |  |
| 0.5-1 | 67 (37.00%) | 55 (42.30%) | 3 (15.80%) | 9 (28.10%) |  |
| 1.5-2 | 15 (8.29%) | 10 (7.69%) | 1 (5.26%) | 4 (12.50%) |  |
| other | 67 (37.00%) | 42 (32.30%) | 13 (68.40%) | 12 (37.50%) |  |

*Represents uncertainty about specific dosages taken by patients.

**Additional Table 3: Comparison of the risk of COVID-19 symptoms in LAM patients by oxygen saturation group^*^**

| **Oxygen Saturation** | **90-95%^**^** | | **≤90%^**^** | |
| --- | --- | --- | --- | --- |
|  | **OR(95%CI)** | **p-value** | **OR(95%CI)** | **p-value** |
| fatigue | 0.68 (0.25, 1.81) | 0.439 | 0.69 (0.15, 3.27) | 0.629 |
| fever | 0.80 (0.20, 3.51) | 0.756 | 1.05 (0.13, 22.23) | 0.967 |
| cough | 0.70 (0.22, 2.30) | 0.545 | 1.01 (0.20, 7.78) | 0.989 |
| anorexia | 0.50 (0.15, 1.52) | 0.234 | 0.50 (0.07, 2.84) | 0.455 |
| dyspnea | 1.71 (0.57, 5.04) | 0.333 | 9.45 (1.96, 56.39) | 0.007 |
| headache | 0.90 (0.34, 2.43) | 0.838 | 2.32 (0.46, 17.49) | 0.341 |
| ageusia | 0.68 (0.18, 2.27) | 0.545 | 1.12 (0.19, 5.34) | 0.892 |
| anosmia | 1.23 (0.44, 3.37) | 0.684 | 0.70 (0.09, 3.49) | 0.688 |
| diarrhoea | 1.36 (0.44, 4.07) | 0.580 | 0.32 (0.02, 2.16) | 0.319 |
| vomiting | 2.07 (0.52, 8.26) | 0.295 | 2.25 (0.28, 13.54) | 0.391 |
| chest pain | 1.05 (0.29, 3.51) | 0.935 | 1.00 (0.14, 5.80) | 0.996 |
| sweats | 0.95 (0.21, 3.80) | 0.946 | 2.76 (0.46, 14.77) | 0.237 |
| myalgia | 1.54 (0.59, 4.05) | 0.376 | 2.35 (0.53, 12.53) | 0.276 |
| sore throat | 1.44 (0.51, 4.31) | 0.498 | 1.01 (0.22, 4.98) | 0.990 |
| hoarse throat | 0.30 (0.09, 0.84) | 0.029 | 2.82 (0.63, 15.77) | 0.194 |
| other | 0.53 (0.01, 8.34) | 0.693 | 0 (NA, Inf) | 0.998 |

*Corrected for confounders of patient age, BMI, co-morbidities, and number of vaccination doses.

**Compared with patients with oxygen saturation levels of 96-100%.

**Additional Table 4: Reasons for Hesitation about COVID-19 Vaccine in LAM Patients**

|  | LAM Patients (N=41) |
| --- | --- |
| **Reasons for Hesitation about COVID-19 Vaccine** |  |
| Worried that the vaccine would aggravate LAM | 65.9%(27/41) |
| Worried about the interaction between vaccine and therapeutic drugs | 36.6%(15/41) |
| Concerned that LAM affects the safety of the vaccine | 24.4%(10/41) |
| Don't think vaccination is necessary | 4.9%(2/41) |
| Doctor did not recommend vaccination for LAM patients | 7.3%(3/41) |
| Just waiting | 9.8%(4/41) |
| Other ^*^ | 14,6%(6/41) |

*Includes "recent surgery", "poor personal health", "suffering from respiratory failure", "preparing for pregnancy", other medical reasons, and "chronic eczema".

**Additional Table 5: Occurrence of Adverse Reactions in the Vaccination Population**

|  | **[ALL]**  ***N=283*** | **HC**  ***N=143*** | **LAM**  ***N=140*** | **p.overall** |
| --- | --- | --- | --- | --- |
| Agee mean(SD) | 42.8 (9.55) | 42.8 (9.08) | 42.8 (10.0) | 0.993 |
| Age stratification: |  |  |  | 0.143 |
| 19~40 | 102 (36.0%) | 44 (30.8%) | 58 (41.4%) |  |
| 40~60 | 170 (60.1%) | 94 (65.7%) | 76 (54.3%) |  |
| >=60 | 11 (3.89%) | 5 (3.50%) | 6 (4.29%) |  |
| BMI stratification: |  |  |  | **<0.001** |
| <18.5 | 27 (9.54%) | 2 (1.40%) | 25 (17.9%) |  |
| 18.5~23.9 | 195 (68.9%) | 96 (67.1%) | 99 (70.7%) |  |
| >=23.9 | 60 (21.2%) | 45 (31.5%) | 15 (10.7%) |  |
| 'Missing' | 1 (0.35%) | 0 (0.00%) | 1 (0.71%) |  |
| Smoking: |  |  |  | 0.367 |
| No | 279 (98.6%) | 142 (99.3%) | 137 (97.9%) |  |
| Yes | 4 (1.41%) | 1 (0.70%) | 3 (2.14%) |  |
| Underlying disease: |  |  |  | **0.007** |
| No | 245 (86.6%) | 132 (92.3%) | 113 (80.7%) |  |
| Yes | 38 (13.4%) | 11 (7.69%) | 27 (19.3%) |  |
| Hypertension: |  |  |  | **0.025** |
| No | 255 (90.1%) | 135 (94.4%) | 120 (85.7%) |  |
| Yes | 28 (9.89%) | 8 (5.59%) | 20 (14.3%) |  |
| Coronary heart disease: |  |  |  | 0.620 |
| No | 280 (98.9%) | 142 (99.3%) | 138 (98.6%) |  |
| Yes | 3 (1.06%) | 1 (0.70%) | 2 (1.43%) |  |
| Diabetes: |  |  |  | 0.120 |
| No | 280 (98.9%) | 143 (100%) | 137 (97.9%) |  |
| Yes | 3 (1.06%) | 0 (0.00%) | 3 (2.14%) |  |
| Tumor: |  |  |  | 1.000 |
| No | 279 (98.6%) | 141 (98.6%) | 138 (98.6%) |  |
| Yes | 4 (1.41%) | 2 (1.40%) | 2 (1.43%) |  |
| Renal insufficiency: No | 283 (100%) | 143 (100%) | 140 (100%) | . |
| Autoimmune disease:: |  |  |  | 0.120 |
| No | 280 (98.9%) | 143 (100%) | 137 (97.9%) |  |
| Yes | 3 (1.06%) | 0 (0.00%) | 3 (2.14%) |  |
| Vaccine dose: |  |  |  | 0.003 |
| 1dose | 8 (2.83%) | 3 (2.10%) | 5 (3.57%) |  |
| 2dose | 44 (15.5%) | 13 (9.09%) | 31 (22.1%) |  |
| 3dose | 220 (77.7%) | 118 (82.5%) | 102 (72.9%) |  |
| 4dose | 11 (3.89%) | 9 (6.29%) | 2 (1.43%) |  |
| Vaccine Type: |  |  |  | <0.001 |
| Inactivated Vaccine | 267 (94.3%) | 143 (100%) | 124 (88.6%) |  |
| Other vaccines | 16 (5.65%) | 0 (0.00%) | 16 (11.4%) |  |
| Type of vaccine for  the first dose: |  |  |  | <0.001 |
| Inactivated vaccines | 272 (96.1%) | 143 (100%) | 129 (92.1%) |  |
| Viral vector vaccine | 5 (1.77%) | 0 (0.00%) | 5 (3.57%) |  |
| Recombinant protein vaccines | 6 (2.12%) | 0 (0.00%) | 6 (4.29%) |  |
| Type of vaccine for  the second dose: |  |  |  | 0.008 |
| Unvaccinated | 8 (2.83%) | 3 (2.10%) | 5 (3.57%) |  |
| Inactivated vaccines | 266 (94.0%) | 140 (97.9%) | 126 (90.0%) |  |
| Viral vector vaccine | 3 (1.06%) | 0 (0.00%) | 3 (2.14%) |  |
| Recombinant protein vaccines | 6 (2.12%) | 0 (0.00%) | 6 (4.29%) |  |
| Type of vaccine for  the third dose: |  |  |  | <0.001 |
| Unvaccinated | 52 (18.4%) | 16 (11.2%) | 36 (25.7%) |  |
| Inactivated vaccines | 221 (78.1%) | 127 (88.8%) | 94 (67.1%) |  |
| Viral vector vaccine | 2 (0.71%) | 0 (0.00%) | 2 (1.43%) |  |
| Recombinant protein vaccines | 8 (2.83%) | 0 (0.00%) | 8 (5.71%) |  |
| Type of vaccine for  the fourth dose: |  |  |  | 0.003 |
| Unvaccinated | 272 (96.1%) | 134 (93.7%) | 138 (98.6%) |  |
| Inactivated vaccines | 9 (3.18%) | 9 (6.29%) | 0 (0.00%) |  |
| Viral vector vaccine | 1 (0.35%) | 0 (0.00%) | 1 (0.71%) |  |
| Recombinant protein vaccines | 1 (0.35%) | 0 (0.00%) | 1 (0.71%) |  |
| Vaccine adverse reactions: |  |  |  | 0.809 |
| No | 241 (85.2%) | 123 (86.0%) | 118 (84.3%) |  |
| Yes | 42 (14.8%) | 20 (14.0%) | 22 (15.7%) |  |
| Redness and swelling(inflamed): |  |  |  | 0.971 |
| No | 272 (96.1%) | 138 (96.5%) | 134 (95.7%) |  |
| Yes | 11 (3.89%) | 5 (3.50%) | 6 (4.29%) |  |
| Induration: |  |  |  | 0.723 |
| No | 275 (97.2%) | 138 (96.5%) | 137 (97.9%) |  |
| Yes | 8 (2.83%) | 5 (3.50%) | 3 (2.14%) |  |
| Local pain: |  |  |  | 0.614 |
| No | 262 (92.6%) | 134 (93.7%) | 128 (91.4%) |  |
| Yes | 21 (7.42%) | 9 (6.29%) | 12 (8.57%) |  |
| Fever: |  |  |  | 1.000 |
| No | 277 (97.9%) | 140 (97.9%) | 137 (97.9%) |  |
| Yes | 6 (2.12%) | 3 (2.10%) | 3 (2.14%) |  |
| Fatigue: |  |  |  | 0.966 |
| No | 268 (94.7%) | 136 (95.1%) | 132 (94.3%) |  |
| Yes | 15 (5.30%) | 7 (4.90%) | 8 (5.71%) |  |
| Nausea, vomiting: |  |  |  | 0.495 |
| No | 282 (99.6%) | 143 (100%) | 139 (99.3%) |  |
| Yes | 1 (0.35%) | 0 (0.00%) | 1 (0.71%) |  |
| Headache: |  |  |  | 0.447 |
| No | 276 (97.5%) | 138 (96.5%) | 138 (98.6%) |  |
| Yes | 7 (2.47%) | 5 (3.50%) | 2 (1.43%) |  |
| Myalgia: |  |  |  | 0.962 |
| No | 264 (93.3%) | 134 (93.7%) | 130 (92.9%) |  |
| Yes | 19 (6.71%) | 9 (6.29%) | 10 (7.14%) |  |
| Diarrhea: |  |  |  | 1.000 |
| No | 282 (99.6%) | 142 (99.3%) | 140 (100%) |  |
| Yes | 1 (0.35%) | 1 (0.70%) | 0 (0.00%) |  |
| Arthralgia: |  |  |  | 0.622 |
| No | 279 (98.6%) | 140 (97.9%) | 139 (99.3%) |  |
| Yes | 4 (1.41%) | 3 (2.10%) | 1 (0.71%) |  |

**Additional Table 6: Subgroup analysis of LAM patients with or without adverse reactions after COVID-19 vaccination**

|  | **[ALL]**  ***N=140*** | **No adverse reactions**  ***N=118*** | **Adverse reactions occurred**  ***N=22*** | **p.overall** |
| --- | --- | --- | --- | --- |
| Age Mean (SD) | 42.8 (10.0) | 43.1 (10.1) | 41.5 (9.99) | 0.486 |
| Age stratification: |  |  |  | 1.000 |
| 19~40 | 58 (41.4%) | 49 (41.5%) | 9 (40.9%) |  |
| 40~60 | 76 (54.3%) | 64 (54.2%) | 12 (54.5%) |  |
| >=60 | 6 (4.29%) | 5 (4.24%) | 1 (4.55%) |  |
| BMI stratification: |  |  |  | 0.382 |
| <18.5 | 25 (17.9%) | 20 (16.9%) | 5 (22.7%) |  |
| 18.5~23.9 | 99 (70.7%) | 86 (72.9%) | 13 (59.1%) |  |
| >=23.9 | 15 (10.7%) | 11 (9.32%) | 4 (18.2%) |  |
| 'Missing' | 1 (0.71%) | 1 (0.85%) | 0 (0.00%) |  |
| Smoking: |  |  |  | 1.000 |
| No | 137 (97.9%) | 115 (97.5%) | 22 (100%) |  |
| Yes | 3 (2.14%) | 3 (2.54%) | 0 (0.00%) |  |
| Underlying disease: |  |  |  | 0.768 |
| No | 113 (80.7%) | 96 (81.4%) | 17 (77.3%) |  |
| Yes | 27 (19.3%) | 22 (18.6%) | 5 (22.7%) |  |
| Hypertension: |  |  |  | 0.200 |
| No | 120 (85.7%) | 99 (83.9%) | 21 (95.5%) |  |
| Yes | 20 (14.3%) | 19 (16.1%) | 1 (4.55%) |  |
| Coronary heart disease: |  |  |  | 1.000 |
| No | 138 (98.6%) | 116 (98.3%) | 22 (100%) |  |
| Yes | 2 (1.43%) | 2 (1.69%) | 0 (0.00%) |  |
| Diabetes: |  |  |  | 0.404 |
| No | 137 (97.9%) | 116 (98.3%) | 21 (95.5%) |  |
| Yes | 3 (2.14%) | 2 (1.69%) | 1 (4.55%) |  |
| Tumor: |  |  |  | 0.291 |
| No | 138 (98.6%) | 117 (99.2%) | 21 (95.5%) |  |
| Yes | 2 (1.43%) | 1 (0.85%) | 1 (4.55%) |  |
| Autoimmune disease: |  |  |  | 0.064 |
| No | 137 (97.9%) | 117 (99.2%) | 20 (90.9%) |  |
| Yes | 3 (2.14%) | 1 (0.85%) | 2 (9.09%) |  |
| Tuberous sclerosis: |  |  |  | 0.929 |
| S-LAM | 99 (70.7%) | 84 (71.2%) | 15 (68.2%) |  |
| TSC-LAM | 13 (9.29%) | 11 (9.32%) | 2 (9.09%) |  |
| No | 28 (20.0%) | 23 (19.5%) | 5 (22.7%) |  |
| Vaccine dose: |  |  |  | 0.417 |
| 1dose | 5 (3.57%) | 4 (3.39%) | 1 (4.55%) |  |
| 2dose | 31 (22.1%) | 26 (22.0%) | 5 (22.7%) |  |
| 3dose | 102 (72.9%) | 87 (73.7%) | 15 (68.2%) |  |
| 4dose | 2 (1.43%) | 1 (0.85%) | 1 (4.55%) |  |
| Vaccine Type: |  |  |  | 0.281 |
| Inactivated Vaccine | 124 (88.6%) | 106 (89.8%) | 18 (81.8%) |  |
| Other vaccines | 16 (11.4%) | 12 (10.2%) | 4 (18.2%) |  |
| Maximum fever temperature*  Median [Q1; Q3] | 38.7 [38.4;39.2] | 38.7 [38.3;39.2] | 39.0 [38.5;39.4] | 0.179 |
| Number of days with a fever higher than 38 degrees# Median [Q1; Q3] | 2.00 [1.00;3.00] | 2.00 [0.00;3.00] | 2.00 [1.00;3.50] | 0.047 |
| Sirolimus or everolimus: |  |  |  | 0.229 |
| No | 25 (17.9%) | 19 (16.1%) | 6 (27.3%) |  |
| Yes | 115 (82.1%) | 99 (83.9%) | 16 (72.7%) |  |
| Whether or not to take sirolimus before or after vaccination: |  |  |  | 0.338 |
| No | 48 (34.3%) | 38 (32.2%) | 10 (45.5%) |  |
| Yes | 92 (65.7%) | 80 (67.8%) | 12 (54.5%) |  |

*Temperature collected only for febrile patients, N=39; #Duration of fever collected only for hyperthermic patients, N=97

**Additional Table 7: Basic information and symptoms of patients with COVID-19 and** **vaccination**

|  | **[ALL]**  ***N=247*** | **HC**  ***N=143*** | **LAM**  ***N=104*** | **p.overall** |
| --- | --- | --- | --- | --- |
| Agee mean(SD) | 42.3 (9.47) | 42.8 (9.08) | 41.6 (9.97) | 0.303 |
| Age stratification: |  |  |  | **0.029** |
| 19~40 | 92 (37.2%) | 44 (30.8%) | 48 (46.2%) |  |
| 40~60 | 145 (58.7%) | 94 (65.7%) | 51 (49.0%) |  |
| >=60 | 10 (4.05%) | 5 (3.50%) | 5 (4.81%) |  |
| BMI |  |  |  | **<0.001** |
| BMI stratification: | 20 (8.10%) | 2 (1.40%) | 18 (17.3%) |  |
| <18.5 | 171 (69.2%) | 96 (67.1%) | 75 (72.1%) |  |
| 18.5~23.9 | 55 (22.3%) | 45 (31.5%) | 10 (9.62%) |  |
| >=23.9 | 1 (0.40%) | 0 (0.00%) | 1 (0.96%) |  |
| 'Missing' |  |  |  | 0.574 |
| Smoking: | 244 (98.8%) | 142 (99.3%) | 102 (98.1%) |  |
| No | 3 (1.21%) | 1 (0.70%) | 2 (1.92%) |  |
| Yes |  |  |  | **0.007** |
| Underlying disease: | 215 (87.0%) | 132 (92.3%) | 83 (79.8%) |  |
| No | 32 (13.0%) | 11 (7.69%) | 21 (20.2%) |  |
| Yes |  |  |  | 0.055 |
| Hypertension: | 225 (91.1%) | 135 (94.4%) | 90 (86.5%) |  |
| No | 22 (8.91%) | 8 (5.59%) | 14 (13.5%) |  |
| Yes |  |  |  | 0.574 |
| Coronary heart disease: | 244 (98.8%) | 142 (99.3%) | 102 (98.1%) |  |
| No | 3 (1.21%) | 1 (0.70%) | 2 (1.92%) |  |
| Yes |  |  |  | 0.176 |
| Diabetes: | 245 (99.2%) | 143 (100%) | 102 (98.1%) |  |
| No | 2 (0.81%) | 0 (0.00%) | 2 (1.92%) |  |
| Tumor: |  |  |  | 1.000 |
| No | 243 (98.4%) | 141 (98.6%) | 102 (98.1%) |  |
| Yes | 4 (1.62%) | 2 (1.40%) | 2 (1.92%) |  |
| Renal insufficiency: | 247 (100%) | 143 (100%) | 104 (100%) | . |
| No |  |  |  | 0.073 |
| Yes | 244 (98.8%) | 143 (100%) | 101 (97.1%) |  |
| Autoimmune disease: | 3 (1.21%) | 0 (0.00%) | 3 (2.88%) |  |
| Vaccine dose: |  |  |  | 0.008 |
| 1dose | 6 (2.43%) | 3 (2.10%) | 3 (2.88%) |  |
| 2dose | 37 (15.0%) | 13 (9.09%) | 24 (23.1%) |  |
| 3dose | 193 (78.1%) | 118 (82.5%) | 75 (72.1%) |  |
| 4dose | 11 (4.45%) | 9 (6.29%) | 2 (1.92%) |  |
| Vaccine Type: |  |  |  | <0.001 |
| Inactivated Vaccine | 236 (95.5%) | 143 (100%) | 93 (89.4%) |  |
| Other vaccines | 11 (4.45%) | 0 (0.00%) | 11 (10.6%) |  |
| Type of vaccine for  the first dose: |  |  |  | 0.005 |
| Inactivated vaccines | 241 (97.6%) | 143 (100%) | 98 (94.2%) |  |
| Viral vector vaccine | 2 (0.81%) | 0 (0.00%) | 2 (1.92%) |  |
| Recombinant protein vaccines | 4 (1.62%) | 0 (0.00%) | 4 (3.85%) |  |
| Type of vaccine for  the second dose: |  |  |  | 0.049 |
| Unvaccinated | 6 (2.43%) | 3 (2.10%) | 3 (2.88%) |  |
| Inactivated vaccines | 237 (96.0%) | 140 (97.9%) | 97 (93.3%) |  |
| Viral vector vaccine | 4 (1.62%) | 0 (0.00%) | 4 (3.85%) |  |
| Recombinant protein vaccines |  |  |  | <0.001 |
| Type of vaccine for  the third dose: | 43 (17.4%) | 16 (11.2%) | 27 (26.0%) |  |
| Unvaccinated | 197 (79.8%) | 127 (88.8%) | 70 (67.3%) |  |
| Inactivated vaccines | 1 (0.40%) | 0 (0.00%) | 1 (0.96%) |  |
| Viral vector vaccine | 6 (2.43%) | 0 (0.00%) | 6 (5.77%) |  |
| Recombinant protein vaccines |  |  |  | 0.005 |
| Type of vaccine for  the fourth dose: | 236 (95.5%) | 134 (93.7%) | 102 (98.1%) |  |
| Unvaccinated | 9 (3.64%) | 9 (6.29%) | 0 (0.00%) |  |
| Inactivated vaccines | 1 (0.40%) | 0 (0.00%) | 1 (0.96%) |  |
| Viral vector vaccine | 1 (0.40%) | 0 (0.00%) | 1 (0.96%) |  |
| Vaccine adverse reactions: |  |  |  | 0.352 |
| No | 207 (83.8%) | 123 (86.0%) | 84 (80.8%) |  |
| Yes | 40 (16.2%) | 20 (14.0%) | 20 (19.2%) |  |
| Lack of power: |  |  |  | <0.001 |
| No | 66 (26.7%) | 23 (16.1%) | 43 (41.3%) |  |
| Yes | 181 (73.3%) | 120 (83.9%) | 61 (58.7%) |  |
| Fever: |  |  |  | 0.106 |
| No | 59 (23.9%) | 40 (28.0%) | 19 (18.3%) |  |
| Yes | 188 (76.1%) | 103 (72.0%) | 85 (81.7%) |  |
| Cough: |  |  |  | 0.881 |
| No | 45 (18.2%) | 27 (18.9%) | 18 (17.3%) |  |
| Yes | 202 (81.8%) | 116 (81.1%) | 86 (82.7%) |  |
| Anorexia: |  |  |  | 0.062 |
| No | 168 (68.0%) | 90 (62.9%) | 78 (75.0%) |  |
| Yes | 79 (32.0%) | 53 (37.1%) | 26 (25.0%) |  |
| Difficulty breathing: |  |  |  | 0.001 |
| No | 208 (84.2%) | 130 (90.9%) | 78 (75.0%) |  |
| Yes | 39 (15.8%) | 13 (9.09%) | 26 (25.0%) |  |
| Headache: |  |  |  | 0.314 |
| No | 106 (42.9%) | 57 (39.9%) | 49 (47.1%) |  |
| Yes | 141 (57.1%) | 86 (60.1%) | 55 (52.9%) |  |
| Ageusia: |  |  |  | 0.123 |
| No | 181 (73.3%) | 99 (69.2%) | 82 (78.8%) |  |
| Yes | 66 (26.7%) | 44 (30.8%) | 22 (21.2%) |  |
| Abnormal sense of smell: |  |  |  | 0.367 |
| No | 175 (70.9%) | 105 (73.4%) | 70 (67.3%) |  |
| Yes | 72 (29.1%) | 38 (26.6%) | 34 (32.7%) |  |
| Diarrhea: |  |  |  | 0.171 |
| No | 201 (81.4%) | 121 (84.6%) | 80 (76.9%) |  |
| Yes | 46 (18.6%) | 22 (15.4%) | 24 (23.1%) |  |
| Vomiting: |  |  |  | 0.573 |
| No | 216 (87.4%) | 127 (88.8%) | 89 (85.6%) |  |
| Yes | 31 (12.6%) | 16 (11.2%) | 15 (14.4%) |  |
| Chest pain: |  |  |  | 0.086 |
| No | 218 (88.3%) | 131 (91.6%) | 87 (83.7%) |  |
| Yes | 29 (11.7%) | 12 (8.39%) | 17 (16.3%) |  |
| Night sweats: |  |  |  | 0.072 |
| No | 189 (76.5%) | 103 (72.0%) | 86 (82.7%) |  |
| Yes | 58 (23.5%) | 40 (28.0%) | 18 (17.3%) |  |
| Myalgia: |  |  |  | 0.620 |
| No | 132 (53.4%) | 74 (51.7%) | 58 (55.8%) |  |
| Yes | 115 (46.6%) | 69 (48.3%) | 46 (44.2%) |  |
| Sore throat: |  |  |  | 0.121 |
| No | 108 (43.7%) | 69 (48.3%) | 39 (37.5%) |  |
| Yes | 139 (56.3%) | 74 (51.7%) | 65 (62.5%) |  |
| Hoarse throat: |  |  |  | 0.450 |
| No | 132 (53.4%) | 73 (51.0%) | 59 (56.7%) |  |
| Yes | 115 (46.6%) | 70 (49.0%) | 45 (43.3%) |  |
| Other or no significant discomfort: |  |  |  | 0.031 |
| No | 225 (91.1%) | 125 (87.4%) | 100 (96.2%) |  |
| Yes | 22 (8.91%) | 18 (12.6%) | 4 (3.85%) |  |
| No significant discomfort: |  |  |  | 0.244 |
| No | 240 (97.2%) | 137 (95.8%) | 103 (99.0%) |  |
| Yes | 7 (2.83%) | 6 (4.20%) | 1 (0.96%) |  |
| Maximum fever temperature*  Median [Q1; Q3] | 38.5 [38.1;39.0] | 38.5 [38.0;39.0] | 38.7 [38.4;39.2] | 0.235 |
| Number of days with a fever higher than 38 degrees# Median [Q1; Q3] | 2.00 [1.00;3.00] | 2.00 [1.00;2.00] | 2.00 [1.00;3.00] | 0.044 |

*N=138 #N=185

**Additional Table 8 Basic information and symptom occurrence in patients with LAM**

|  | **[ALL]**  ***N=131*** | **Unvaccinated**  ***N=27*** | **Vaccinated *N=104*** | **p.overall** |
| --- | --- | --- | --- | --- |
| Agee mean(SD) | 42.3 (10.0) | 45.0 (9.99) | 41.6 (9.97) | 0.123 |
| Age stratification: |  |  |  | 0.589 |
| 19~40 | 58 (44.3%) | 10 (37.0%) | 48 (46.2%) |  |
| 40~60 | 66 (50.4%) | 15 (55.6%) | 51 (49.0%) |  |
| >=60 | 7 (5.34%) | 2 (7.41%) | 5 (4.81%) |  |
| BMI |  |  |  | 0.143 |
| BMI stratification: | 26 (19.8%) | 8 (29.6%) | 18 (17.3%) |  |
| <18.5 | 89 (67.9%) | 14 (51.9%) | 75 (72.1%) |  |
| 18.5~23.9 | 14 (10.7%) | 4 (14.8%) | 10 (9.62%) |  |
| >=23.9 | 2 (1.53%) | 1 (3.70%) | 1 (0.96%) |  |
| 'Missing' |  |  |  | 0.503 |
| Smoking: | 128 (97.7%) | 26 (96.3%) | 102 (98.1%) |  |
| No | 3 (2.29%) | 1 (3.70%) | 2 (1.92%) |  |
| Yes |  |  |  | 0.701 |
| Underlying disease: | 103 (78.6%) | 20 (74.1%) | 83 (79.8%) |  |
| No | 28 (21.4%) | 7 (25.9%) | 21 (20.2%) |  |
| Yes |  |  |  | 1.000 |
| Hypertension: | 113 (86.3%) | 23 (85.2%) | 90 (86.5%) |  |
| No | 18 (13.7%) | 4 (14.8%) | 14 (13.5%) |  |
| Yes |  |  |  | 1.000 |
| Coronary heart disease: | 129 (98.5%) | 27 (100%) | 102 (98.1%) |  |
| No | 2 (1.53%) | 0 (0.00%) | 2 (1.92%) |  |
| Yes |  |  |  | 0.503 |
| Diabetes: | 128 (97.7%) | 26 (96.3%) | 102 (98.1%) |  |
| No | 3 (2.29%) | 1 (3.70%) | 2 (1.92%) |  |
| Tumor: |  |  |  | 0.188 |
| No | 127 (96.9%) | 25 (92.6%) | 102 (98.1%) |  |
| Yes | 4 (3.05%) | 2 (7.41%) | 2 (1.92%) |  |
| Renal insufficiency: |  |  |  | 0.206 |
| No | 130 (99.2%) | 26 (96.3%) | 104 (100%) |  |
| Yes | 1 (0.76%) | 1 (3.70%) | 0 (0.00%) |  |
| Autoimmune disease: |  |  |  | 1.000 |
| No | 127 (96.9%) | 26 (96.3%) | 101 (97.1%) |  |
| Yes | 4 (3.05%) | 1 (3.70%) | 3 (2.88%) |  |
| Vaccine dose: |  |  |  | <0.001 |
| 1dose | 3 (2.29%) | 0 (0.00%) | 3 (2.88%) |  |
| 2dose | 24 (18.3%) | 0 (0.00%) | 24 (23.1%) |  |
| 3dose | 75 (57.3%) | 0 (0.00%) | 75 (72.1%) |  |
| 4dose | 2 (1.53%) | 0 (0.00%) | 2 (1.92%) |  |
| 'Missing' | 27 (20.6%) | 27 (100%) | 0 (0.00%) |  |
| Type of vaccine for  the fourth dose: |  |  |  | <0.001 |
| Unvaccinated | 27 (20.6%) | 27 (100%) | 0 (0.00%) |  |
| Inactivated vaccines | 93 (71.0%) | 0 (0.00%) | 93 (89.4%) |  |
| Other vaccines | 11 (8.40%) | 0 (0.00%) | 11 (10.6%) |  |
| Type of vaccine for  the first dose: |  |  |  | <0.001 |
| Unvaccinated | 27 (20.6%) | 27 (100%) | 0 (0.00%) |  |
| Inactivated vaccines | 98 (74.8%) | 0 (0.00%) | 98 (94.2%) |  |
| Viral vector vaccine | 2 (1.53%) | 0 (0.00%) | 2 (1.92%) |  |
| Recombinant protein vaccines | 4 (3.05%) | 0 (0.00%) | 4 (3.85%) |  |
| Type of vaccine for  the second dose: |  |  |  | <0.001 |
| Unvaccinated | 30 (22.9%) | 27 (100%) | 3 (2.88%) |  |
| Inactivated vaccines | 97 (74.0%) | 0 (0.00%) | 97 (93.3%) |  |
| Recombinant protein vaccines | 4 (3.05%) | 0 (0.00%) | 4 (3.85%) |  |
| Type of vaccine for  the third dose: |  |  |  | <0.001 |
| Unvaccinated | 54 (41.2%) | 27 (100%) | 27 (26.0%) |  |
| Inactivated vaccines | 70 (53.4%) | 0 (0.00%) | 70 (67.3%) |  |
| Viral vector vaccine | 1 (0.76%) | 0 (0.00%) | 1 (0.96%) |  |
| Recombinant protein vaccines | 6 (4.58%) | 0 (0.00%) | 6 (5.77%) |  |
| Type of vaccine for  the fourth dose: |  |  |  | 1.000 |
| Unvaccinated | 129 (98.5%) | 27 (100%) | 102 (98.1%) |  |
| Inactivated vaccines | 1 (0.76%) | 0 (0.00%) | 1 (0.96%) |  |
| Viral vector vaccine | 1 (0.76%) | 0 (0.00%) | 1 (0.96%) |  |
| Vaccine adverse reactions: |  |  |  | 0.013 |
| No | 111 (84.7%) | 27 (100%) | 84 (80.8%) |  |
| Yes | 20 (15.3%) | 0 (0.00%) | 20 (19.2%) |  |
| Lack of power: |  |  |  | 0.108 |
| No | 49 (37.4%) | 6 (22.2%) | 43 (41.3%) |  |
| Yes | 82 (62.6%) | 21 (77.8%) | 61 (58.7%) |  |
| Fever: |  |  |  | 0.243 |
| No | 21 (16.0%) | 2 (7.41%) | 19 (18.3%) |  |
| Yes | 110 (84.0%) | 25 (92.6%) | 85 (81.7%) |  |
| Cough: |  |  |  | 0.581 |
| No | 24 (18.3%) | 6 (22.2%) | 18 (17.3%) |  |
| Yes | 107 (81.7%) | 21 (77.8%) | 86 (82.7%) |  |
| Anorexia: |  |  |  | <0.001 |
| No | 87 (66.4%) | 9 (33.3%) | 78 (75.0%) |  |
| Yes | 44 (33.6%) | 18 (66.7%) | 26 (25.0%) |  |
| Difficulty breathing: |  |  |  | 0.314 |
| No | 95 (72.5%) | 17 (63.0%) | 78 (75.0%) |  |
| Yes | 36 (27.5%) | 10 (37.0%) | 26 (25.0%) |  |
| Headache: |  |  |  | 0.078 |
| No | 56 (42.7%) | 7 (25.9%) | 49 (47.1%) |  |
| Yes | 75 (57.3%) | 20 (74.1%) | 55 (52.9%) |  |
| Ageusia: |  |  |  | 0.027 |
| No | 97 (74.0%) | 15 (55.6%) | 82 (78.8%) |  |
| Yes | 34 (26.0%) | 12 (44.4%) | 22 (21.2%) |  |
| Abnormal sense of smell: |  |  |  | 0.942 |
| No | 89 (67.9%) | 19 (70.4%) | 70 (67.3%) |  |
| Yes | 42 (32.1%) | 8 (29.6%) | 34 (32.7%) |  |
| Diarrhea: |  |  |  | 0.398 |
| No | 98 (74.8%) | 18 (66.7%) | 80 (76.9%) |  |
| Yes | 33 (25.2%) | 9 (33.3%) | 24 (23.1%) |  |
| Vomiting: |  |  |  | 1.000 |
| No | 112 (85.5%) | 23 (85.2%) | 89 (85.6%) |  |
| Yes | 19 (14.5%) | 4 (14.8%) | 15 (14.4%) |  |
| Chest pain: |  |  |  | 0.089 |
| No | 105 (80.2%) | 18 (66.7%) | 87 (83.7%) |  |
| Yes | 26 (19.8%) | 9 (33.3%) | 17 (16.3%) |  |
| Night sweats: |  |  |  | 0.459 |
| No | 106 (80.9%) | 20 (74.1%) | 86 (82.7%) |  |
| Yes | 25 (19.1%) | 7 (25.9%) | 18 (17.3%) |  |
| Myalgia: |  |  |  | 0.027 |
| No | 66 (50.4%) | 8 (29.6%) | 58 (55.8%) |  |
| Yes | 65 (49.6%) | 19 (70.4%) | 46 (44.2%) |  |
| Sore throat: |  |  |  | 1.000 |
| No | 49 (37.4%) | 10 (37.0%) | 39 (37.5%) |  |
| Yes | 82 (62.6%) | 17 (63.0%) | 65 (62.5%) |  |
| Hoarse throat: |  |  |  | 0.715 |
| No | 76 (58.0%) | 17 (63.0%) | 59 (56.7%) |  |
| Yes | 55 (42.0%) | 10 (37.0%) | 45 (43.3%) |  |
| Other or no significant discomfort: |  |  |  | 0.153 |
| No | 124 (94.7%) | 24 (88.9%) | 100 (96.2%) |  |
| Yes | 7 (5.34%) | 3 (11.1%) | 4 (3.85%) |  |
| No significant discomfort: |  |  |  | 1.000 |
| No | 130 (99.2%) | 27 (100%) | 103 (99.0%) |  |
| Yes | 1 (0.76%) | 0 (0.00%) | 1 (0.96%) |  |
| Maximum fever temperature*  Median [Q1; Q3] | 38.7 [38.3;39.0] | 38.5 [38.0;39.0] | 38.7 [38.4;39.2] | 0.326 |
| Number of days with a fever higher than 38 degrees# Median [Q1; Q3] | 2.00 [1.00;3.00] | 2.00 [0.75;2.00] | 2.00 [1.00;3.00] | 0.266 |

*N=52; #N=107

**Additional Table 9. Vaccination and exacerbation of symptoms in patients with LAM**

|  | **[ALL]**  ***N=131*** | **Unvaccinated**  ***N=27*** | **Vaccinated**  ***N=104*** | **p.overall** |
| --- | --- | --- | --- | --- |
| Agee mean(SD) | 42.3 (10.0) | 45.0 (9.99) | 41.6 (9.97) | 0.123 |
| Age stratification: |  |  |  | 0.589 |
| 19~40 | 58 (44.3%) | 10 (37.0%) | 48 (46.2%) |  |
| 40~60 | 66 (50.4%) | 15 (55.6%) | 51 (49.0%) |  |
| >=60 | 7 (5.34%) | 2 (7.41%) | 5 (4.81%) |  |
| BMI stratification: |  |  |  | 0.143 |
| <18.5 | 26 (19.8%) | 8 (29.6%) | 18 (17.3%) |  |
| 18.5~23.9 | 89 (67.9%) | 14 (51.9%) | 75 (72.1%) |  |
| >=23.9 | 14 (10.7%) | 4 (14.8%) | 10 (9.62%) |  |
| 'Missing' | 2 (1.53%) | 1 (3.70%) | 1 (0.96%) |  |
| Smoking: |  |  |  | 0.503 |
| No | 128 (97.7%) | 26 (96.3%) | 102 (98.1%) |  |
| Yes | 3 (2.29%) | 1 (3.70%) | 2 (1.92%) |  |
| Underlying disease: |  |  |  | 0.701 |
| No | 103 (78.6%) | 20 (74.1%) | 83 (79.8%) |  |
| Yes | 28 (21.4%) | 7 (25.9%) | 21 (20.2%) |  |
| Hypertension: |  |  |  | 1.000 |
| No | 113 (86.3%) | 23 (85.2%) | 90 (86.5%) |  |
| Yes | 18 (13.7%) | 4 (14.8%) | 14 (13.5%) |  |
| Coronary heart disease: |  |  |  | 1.000 |
| No | 129 (98.5%) | 27 (100%) | 102 (98.1%) |  |
| Yes | 2 (1.53%) | 0 (0.00%) | 2 (1.92%) |  |
| Diabetes: |  |  |  | 0.503 |
| No | 128 (97.7%) | 26 (96.3%) | 102 (98.1%) |  |
| Yes | 3 (2.29%) | 1 (3.70%) | 2 (1.92%) |  |
| Tumor: |  |  |  | 0.188 |
| No | 127 (96.9%) | 25 (92.6%) | 102 (98.1%) |  |
| Yes | 4 (3.05%) | 2 (7.41%) | 2 (1.92%) |  |
| Renal insufficiency: |  |  |  | 0.206 |
| No | 130 (99.2%) | 26 (96.3%) | 104 (100%) |  |
| Yes | 1 (0.76%) | 1 (3.70%) | 0 (0.00%) |  |
| Autoimmune disease: |  |  |  | 1.000 |
| No | 127 (96.9%) | 26 (96.3%) | 101 (97.1%) |  |
| Yes | 4 (3.05%) | 1 (3.70%) | 3 (2.88%) |  |
| Sirolimus after SARS-CoV-2 infection: |  |  |  | <0.001 |
| No | 63 (48.1%) | 27 (100%) | 36 (34.6%) |  |
| Yes | 68 (51.9%) | 0 (0.00%) | 68 (65.4%) |  |
| Vaccine dose: |  |  |  | <0.001 |
| 1dose | 3 (2.29%) | 0 (0.00%) | 3 (2.88%) |  |
| 2dose | 24 (18.3%) | 0 (0.00%) | 24 (23.1%) |  |
| 3dose | 75 (57.3%) | 0 (0.00%) | 75 (72.1%) |  |
| 4dose | 2 (1.53%) | 0 (0.00%) | 2 (1.92%) |  |
| 'Missing' | 27 (20.6%) | 27 (100%) | 0 (0.00%) |  |
| Vaccine Type: |  |  |  | <0.001 |
| Unvaccinated | 27 (20.6%) | 27 (100%) | 0 (0.00%) |  |
| Inactivated vaccines | 93 (71.0%) | 0 (0.00%) | 93 (89.4%) |  |
| Viral vector vaccine | 11 (8.40%) | 0 (0.00%) | 11 (10.6%) |  |
| Adverse reaction: |  |  |  | 0.013 |
| No | 111 (84.7%) | 27 (100%) | 84 (80.8%) |  |
| Yes | 20 (15.3%) | 0 (0.00%) | 20 (19.2%) |  |
| No significant change: |  |  |  | 0.728 |
| No | 96 (73.3%) | 21 (77.8%) | 75 (72.1%) |  |
| Yes | 35 (26.7%) | 6 (22.2%) | 29 (27.9%) |  |
| Worse fatigue: |  |  |  | 0.318 |
| No | 67 (51.1%) | 11 (40.7%) | 56 (53.8%) |  |
| Yes | 64 (48.9%) | 16 (59.3%) | 48 (46.2%) |  |
| Worsening of cough: |  |  |  | 0.812 |
| No | 73 (55.7%) | 14 (51.9%) | 59 (56.7%) |  |
| Yes | 58 (44.3%) | 13 (48.1%) | 45 (43.3%) |  |
| Worse dyspnea: |  |  |  | 1.000 |
| No | 93 (71.0%) | 19 (70.4%) | 74 (71.2%) |  |
| Yes | 38 (29.0%) | 8 (29.6%) | 30 (28.8%) |  |
| Worsening of chest tightness: |  |  |  | 1.000 |
| No | 105 (80.2%) | 22 (81.5%) | 83 (79.8%) |  |
| Yes | 26 (19.8%) | 5 (18.5%) | 21 (20.2%) |  |
| Worsening of chest pain: |  |  |  | 1.000 |
| No | 114 (87.0%) | 24 (88.9%) | 90 (86.5%) |  |
| Yes | 17 (13.0%) | 3 (11.1%) | 14 (13.5%) |  |
| Worsening hemoptysis: |  |  |  | 0.633 |
| No | 124 (94.7%) | 25 (92.6%) | 99 (95.2%) |  |
| Yes | 7 (5.34%) | 2 (7.41%) | 5 (4.81%) |  |
| Increased pleural effusion: |  |  |  | 1.000 |
| No | 129 (98.5%) | 27 (100%) | 102 (98.1%) |  |
| Yes | 2 (1.53%) | 0 (0.00%) | 2 (1.92%) |  |
| Development of spontaneous pneumothorax: |  |  |  | 0.371 |
| No | 129 (98.5%) | 26 (96.3%) | 103 (99.0%) |  |
| Yes | 2 (1.53%) | 1 (3.70%) | 1 (0.96%) |  |

**Additional Table 10: Effect of long-term administration of mTOR inhibitors on COVID-19 symptoms in patients with LAM**

|  | **[ALL]**  ***N=93*** | **NmTOR**  ***N=15*** | **mTOR**  ***N=78*** | **p.overall** |
| --- | --- | --- | --- | --- |
| Age: |  |  |  | 0.891 |
| 19~40 | 41 (44.1%) | 6 (40.0%) | 35 (44.9%) |  |
| 40~60 | 48 (51.6%) | 9 (60.0%) | 39 (50.0%) |  |
| >=60 | 4 (4.30%) | 0 (0.00%) | 4 (5.13%) |  |
| BMI: |  |  |  | 0.019 |
| 13.3~18.5 | 17 (18.3%) | 2 (13.3%) | 15 (19.2%) |  |
| 18.5~23.9 | 66 (71.0%) | 8 (53.3%) | 58 (74.4%) |  |
| 23.9~42.5 | 9 (9.68%) | 4 (26.7%) | 5 (6.41%) |  |
| 'Missing' | 1 (1.08%) | 1 (6.67%) | 0 (0.00%) |  |
| Smoking: |  |  |  | 1.000 |
| No | 91 (97.8%) | 15 (100%) | 76 (97.4%) |  |
| Yes | 2 (2.15%) | 0 (0.00%) | 2 (2.56%) |  |
| Underlying disease: |  |  |  | 0.732 |
| No | 73 (78.5%) | 11 (73.3%) | 62 (79.5%) |  |
| Yes | 20 (21.5%) | 4 (26.7%) | 16 (20.5%) |  |
| Hypertension: |  |  |  | 1.000 |
| No | 80 (86.0%) | 13 (86.7%) | 67 (85.9%) |  |
| Yes | 13 (14.0%) | 2 (13.3%) | 11 (14.1%) |  |
| Coronary heart disease: |  |  |  | 1.000 |
| No | 91 (97.8%) | 15 (100%) | 76 (97.4%) |  |
| Yes | 2 (2.15%) | 0 (0.00%) | 2 (2.56%) |  |
| Diabetes: |  |  |  | 1.000 |
| No | 91 (97.8%) | 15 (100%) | 76 (97.4%) |  |
| Yes | 2 (2.15%) | 0 (0.00%) | 2 (2.56%) |  |
| Tumor: |  |  |  | 1.000 |
| No | 91 (97.8%) | 15 (100%) | 76 (97.4%) |  |
| Yes | 2 (2.15%) | 0 (0.00%) | 2 (2.56%) |  |
| Autoimmune disease: |  |  |  | 0.067 |
| No | 90 (96.8%) | 13 (86.7%) | 77 (98.7%) |  |
| Yes | 3 (3.23%) | 2 (13.3%) | 1 (1.28%) |  |
| Vaccine dose: |  |  |  | 0.365 |
| 1dose | 2 (2.15%) | 1 (6.67%) | 1 (1.28%) |  |
| 2dose | 23 (24.7%) | 4 (26.7%) | 19 (24.4%) |  |
| 3dose | 68 (73.1%) | 10 (66.7%) | 58 (74.4%) |  |
| Vaccine adverse reactions: |  |  |  | 0.020 |
| No | 77 (82.8%) | 9 (60.0%) | 68 (87.2%) |  |
| Yes | 16 (17.2%) | 6 (40.0%) | 10 (12.8%) |  |
| Lack of power: |  |  |  | 0.111 |
| No | 39 (41.9%) | 3 (20.0%) | 36 (46.2%) |  |
| Yes | 54 (58.1%) | 12 (80.0%) | 42 (53.8%) |  |
| Fever: |  |  |  | 0.287 |
| No | 18 (19.4%) | 1 (6.67%) | 17 (21.8%) |  |
| Yes | 75 (80.6%) | 14 (93.3%) | 61 (78.2%) |  |
| Cough: |  |  |  | 0.730 |
| No | 17 (18.3%) | 2 (13.3%) | 15 (19.2%) |  |
| Yes | 76 (81.7%) | 13 (86.7%) | 63 (80.8%) |  |
| Anorexia: |  |  |  | 0.048 |
| No | 70 (75.3%) | 8 (53.3%) | 62 (79.5%) |  |
| Yes | 23 (24.7%) | 7 (46.7%) | 16 (20.5%) |  |
| Difficulty breathing: |  |  |  | 0.752 |
| No | 69 (74.2%) | 12 (80.0%) | 57 (73.1%) |  |
| Yes | 24 (25.8%) | 3 (20.0%) | 21 (26.9%) |  |
| Headache: |  |  |  | 0.877 |
| No | 42 (45.2%) | 6 (40.0%) | 36 (46.2%) |  |
| Yes | 51 (54.8%) | 9 (60.0%) | 42 (53.8%) |  |
| Ageusia: |  |  |  | 0.012 |
| No | 74 (79.6%) | 8 (53.3%) | 66 (84.6%) |  |
| Yes | 19 (20.4%) | 7 (46.7%) | 12 (15.4%) |  |
| Abnormal sense of smell: |  |  |  | 0.370 |
| No | 62 (66.7%) | 8 (53.3%) | 54 (69.2%) |  |
| Yes | 31 (33.3%) | 7 (46.7%) | 24 (30.8%) |  |
| Diarrhea: |  |  |  | 0.336 |
| No | 71 (76.3%) | 10 (66.7%) | 61 (78.2%) |  |
| Yes | 22 (23.7%) | 5 (33.3%) | 17 (21.8%) |  |
| Vomiting: |  |  |  | 0.704 |
| No | 78 (83.9%) | 12 (80.0%) | 66 (84.6%) |  |
| Yes | 15 (16.1%) | 3 (20.0%) | 12 (15.4%) |  |
| Chest pain: |  |  |  | 0.062 |
| No | 78 (83.9%) | 10 (66.7%) | 68 (87.2%) |  |
| Yes | 15 (16.1%) | 5 (33.3%) | 10 (12.8%) |  |
| Night sweats: |  |  |  | 0.704 |
| No | 78 (83.9%) | 12 (80.0%) | 66 (84.6%) |  |
| Yes | 15 (16.1%) | 3 (20.0%) | 12 (15.4%) |  |
| Myalgia: |  |  |  | 1.000 |
| No | 51 (54.8%) | 8 (53.3%) | 43 (55.1%) |  |
| Yes | 42 (45.2%) | 7 (46.7%) | 35 (44.9%) |  |
| Sore throat: |  |  |  | 0.859 |
| No | 36 (38.7%) | 5 (33.3%) | 31 (39.7%) |  |
| Yes | 57 (61.3%) | 10 (66.7%) | 47 (60.3%) |  |
| Hoarse throat: |  |  |  | 1.000 |
| No | 50 (53.8%) | 8 (53.3%) | 42 (53.8%) |  |
| Yes | 43 (46.2%) | 7 (46.7%) | 36 (46.2%) |  |
| Other or no significant discomfort: |  |  |  | 1.000 |
| No | 90 (96.8%) | 15 (100%) | 75 (96.2%) |  |
| Yes | 3 (3.23%) | 0 (0.00%) | 3 (3.85%) |  |
| No significant discomfort: |  |  |  | 1.000 |
| No | 92 (98.9%) | 15 (100%) | 77 (98.7%) |  |
| Yes | 1 (1.08%) | 0 (0.00%) | 1 (1.28%) |  |
| Maximum fever temperature | 38.7 (0.55) | 38.7 (0.87) | 38.7 (0.44) | 0.978 |
| Number of days with a fever higher than 38 degrees | 2.00 [1.00;3.00] | 2.00 [1.00;4.25] | 2.00 [1.00;3.00] | 0.306 |

NmTOR：Not taking mTOR inhibitors

**Additional Table 11: COVID-19 Symptoms in Vaccinated LAM Patients versus Healthy Adults**

| **Feature** | **[ALL]**  **N=247** | **HC**  **N=143** | **LAM**  **N=104** | **OR (95%CI)** | **p-value** |
| --- | --- | --- | --- | --- | --- |
| **dyspnea** |  |  |  |  | 0.12 |
| No | 208(84.2%) | 130 (90.9%) | 78 (75.0%) | reference |  |
| Yes | 39(15.8%) | 13 (9.09%) | 26 (25.0%) | 2.08 (0.94,4.6) |  |
|  |  |  |  |  |  |
| **fever** |  |  |  |  | 0.63 |
| No | 59(23.9%) | 40 (28.0%) | 19 (18.3%) | reference |  |
| Yes | 188(76.1%) | 103 (72.0%) | 85 (81.7%) | 1.55 (0.78,3.1) |  |
|  |  |  |  |  |  |
| **sore throat** |  |  |  |  | 0.13 |
| No | 108(43.7%) | 69 (48.3%) | 39 (37.5%) | reference |  |
| Yes | 139(56.3%) | 74 (51.7%) | 65 (62.5%) | 1.53 (0.88,2.65) |  |
|  |  |  |  |  |  |
| **vomiting** |  |  |  |  | 0.33 |
| No | 216(87.4%) | 127 (88.8%) | 89 (85.6%) | reference |  |
| Yes | 31(12.6%) | 16 (11.2%) | 15 (14.4%) | 1.53 (0.65,3.6) |  |
|  |  |  |  |  |  |
| **diarrhoea** |  |  |  |  | 0.3 |
| No | 201(81.4%) | 121 (84.6%) | 80 (76.9%) | reference |  |
| Yes | 46(18.6%) | 22 (15.4%) | 24 (23.1%) | 1.49 (0.7,3.16) |  |
|  |  |  |  |  |  |
| **chest pain** |  |  |  |  | 0.49 |
| No | 218(88.3%) | 131 (91.6%) | 87 (83.7%) | reference |  |
| Yes | 29(11.7%) | 12 (8.39%) | 17 (16.3%) | 1.38 (0.56,3.4) |  |
|  |  |  |  |  |  |
| **anosmia** |  |  |  |  | 0.41 |
| No | 175(70.9%) | 105 (73.4%) | 70 (67.3%) | reference |  |
| Yes | 72(29.1%) | 38 (26.6%) | 34 (32.7%) | 1.24 (0.71,2.18) |  |
|  |  |  |  |  |  |
| **cough** |  |  |  |  | 0.75 |
| No | 45(18.2%) | 27 (18.9%) | 18 (17.3%) | reference |  |
| Yes | 202(81.8%) | 116 (81.1%) | 86 (82.7%) | 1.04 (0.53,2.03) |  |
|  |  |  |  |  |  |
| **hoarse throat** |  |  |  |  | 0.26 |
| No | 132(53.4%) | 73 (51.0%) | 59 (56.7%) | reference |  |
| Yes | 115(46.6%) | 70 (49.0%) | 45 (43.3%) | 0.74 (0.44,1.25) |  |
|  |  |  |  |  |  |
| **Myalgia** |  |  |  |  | 0.25 |
| No | 132(53.4%) | 74 (51.7%) | 58 (55.8%) | reference |  |
| Yes | 115(46.6%) | 69 (48.3%) | 46 (44.2%) | 0.73 (0.43,1.25) |  |
| **headache** |  |  |  |  | 0.18 |
| No | 106(42.9%) | 57 (39.9%) | 49 (47.1%) | reference |  |
| Yes | 141(57.1%) | 86 (60.1%) | 55 (52.9%) | 0.70 (0.42,1.18) |  |
|  |  |  |  |  |  |
| **ageusia** |  |  |  |  | 0.1 |
| No | 181(73.3%) | 99 (69.2%) | 82 (78.8%) | reference |  |
| Yes | 66(26.7%) | 44 (30.8%) | 22 (21.2%) | 0.61 (0.33,1.1) |  |
|  |  |  |  |  |  |
| **sweats** |  |  |  |  | 0.062 |
| No | 189(76.5%) | 103 (72.0%) | 86 (82.7%) | reference |  |
| Yes | 58(23.5%) | 40 (28.0%) | 18 (17.3%) | 0.54 (0.28,1.03) |  |
|  |  |  |  |  |  |
| **anorexia** |  |  |  |  | 0.058 |
| No | 168(68.0%) | 90 (62.9%) | 78 (75.0%) | reference |  |
| Yes | 79(32.0%) | 53 (37.1%) | 26 (25.0%) | 0.48 (0.25,0.9) |  |
|  |  |  |  |  |  |
| **fatigue** |  |  |  |  | **<0.001** |
| No | 66(26.7%) | 23 (16.1%) | 43 (41.3%) | reference |  |
| Yes | 181(73.3%) | 120 (83.9%) | 61 (58.7%) | 0.26 (0.14,0.49) |  |
|  |  |  |  |  |  |
| **other symptom** |  |  |  |  | 0.055 |
| No | 225(91.1%) | 125 (87.4%) | 100 (96.2%) | reference |  |
| Yes | 22(8.9%) | 18 (12.6%) | 4 (3.85%) | 0.32 (0.1,1.02) |  |
