# Supplementary material for: Safety and efficacy of COVID-19 vaccination in the Chinese population with pulmonary lymphangioleiomyomatosis: a single-center retrospective study
Source: Orphanet J Rare Dis. 2024 Jul 3;19:247. doi: 10.1186/s13023-024-03260-4 (PMC11220960; doi:10.1186/s13023-024-03260-4)
Supplement: Supplementary file 4 — Supplementary Material 4 [file 13023_2024_3260_MOESM4_ESM.docx]

**Additional file 4:**


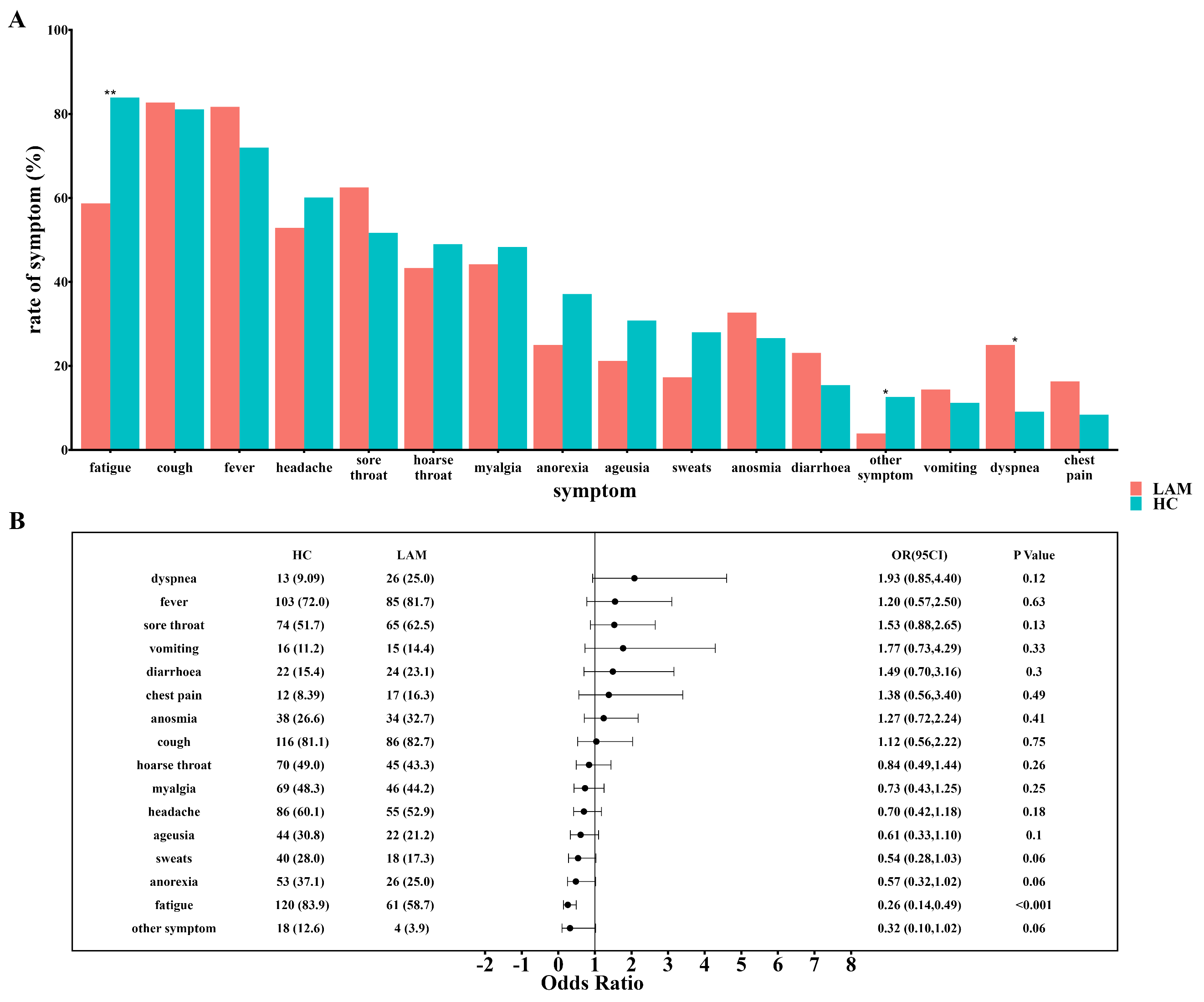


**Additional Figure 1. Risk analysis of COVID-19 symptoms in vaccinated healthy individuals versus LAM patients (n=247)**


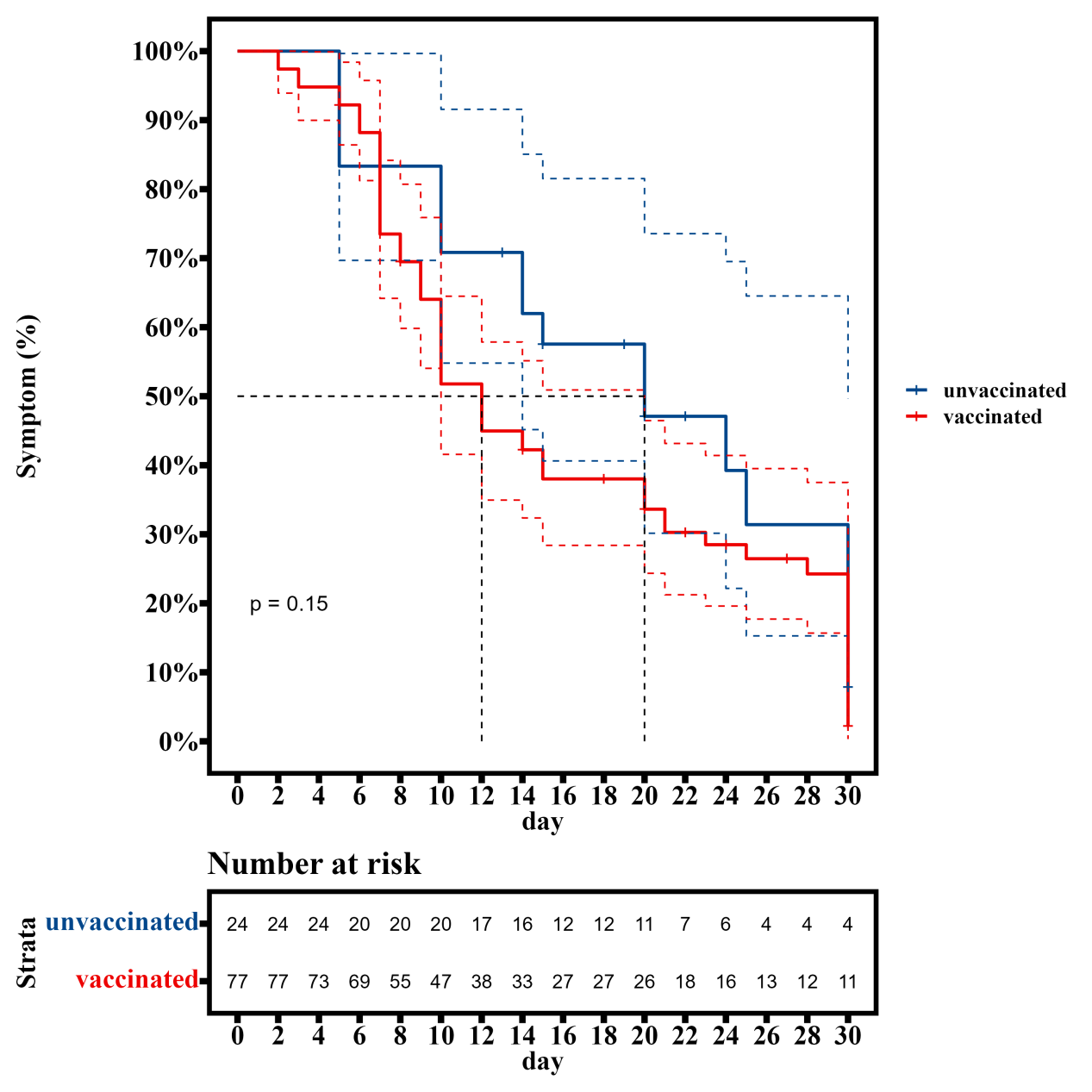


**Additional Figure 2. Duration of symptoms in vaccinated LAM patients vs. unvaccinated LAM patients (N=131)**


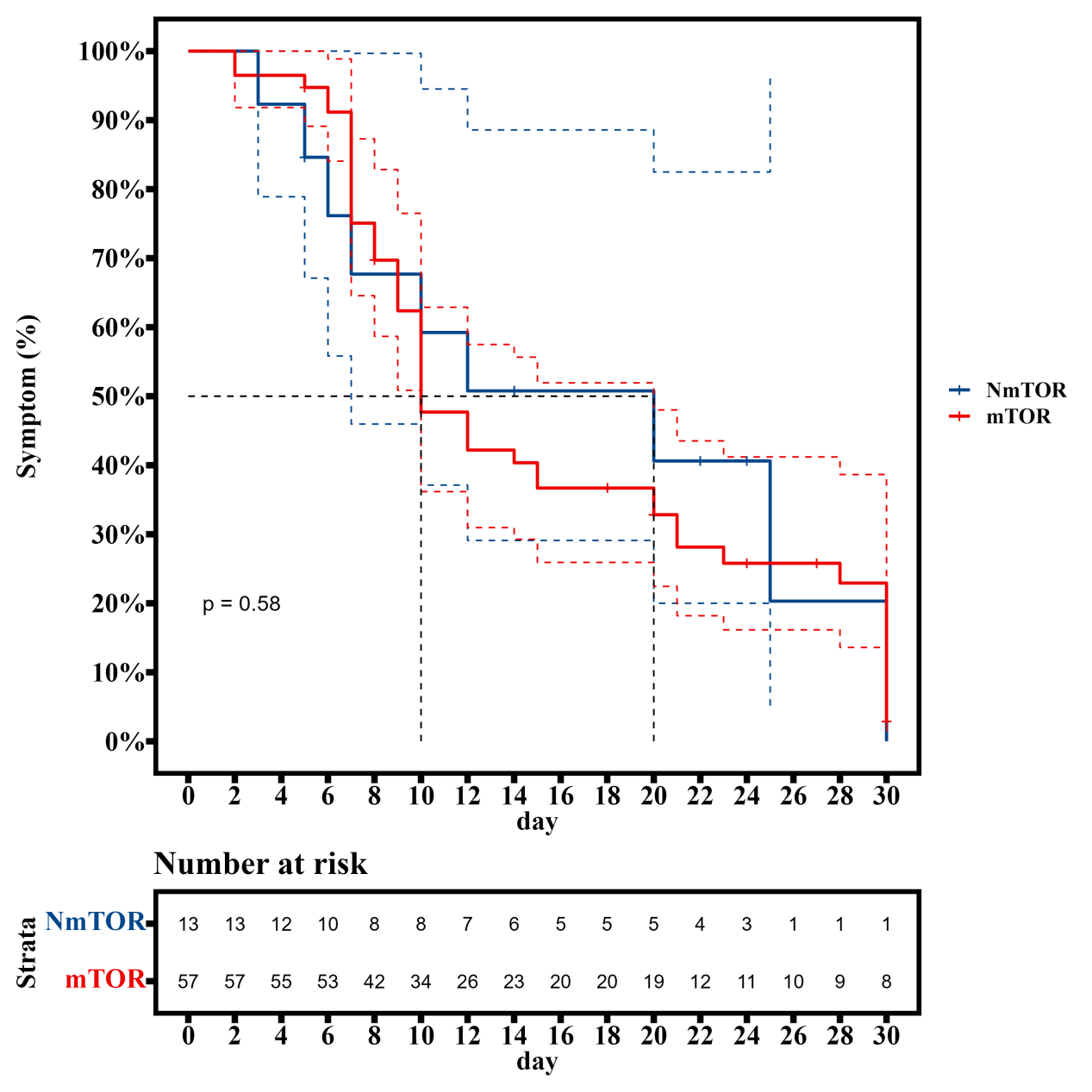


**Additional Figure 3. Duration of Symptoms in LAM Patients with Regular Use of mTOR Inhibitors vs. LAM Patients Without Regular Use of mTOR Inhibitors (N=93)**


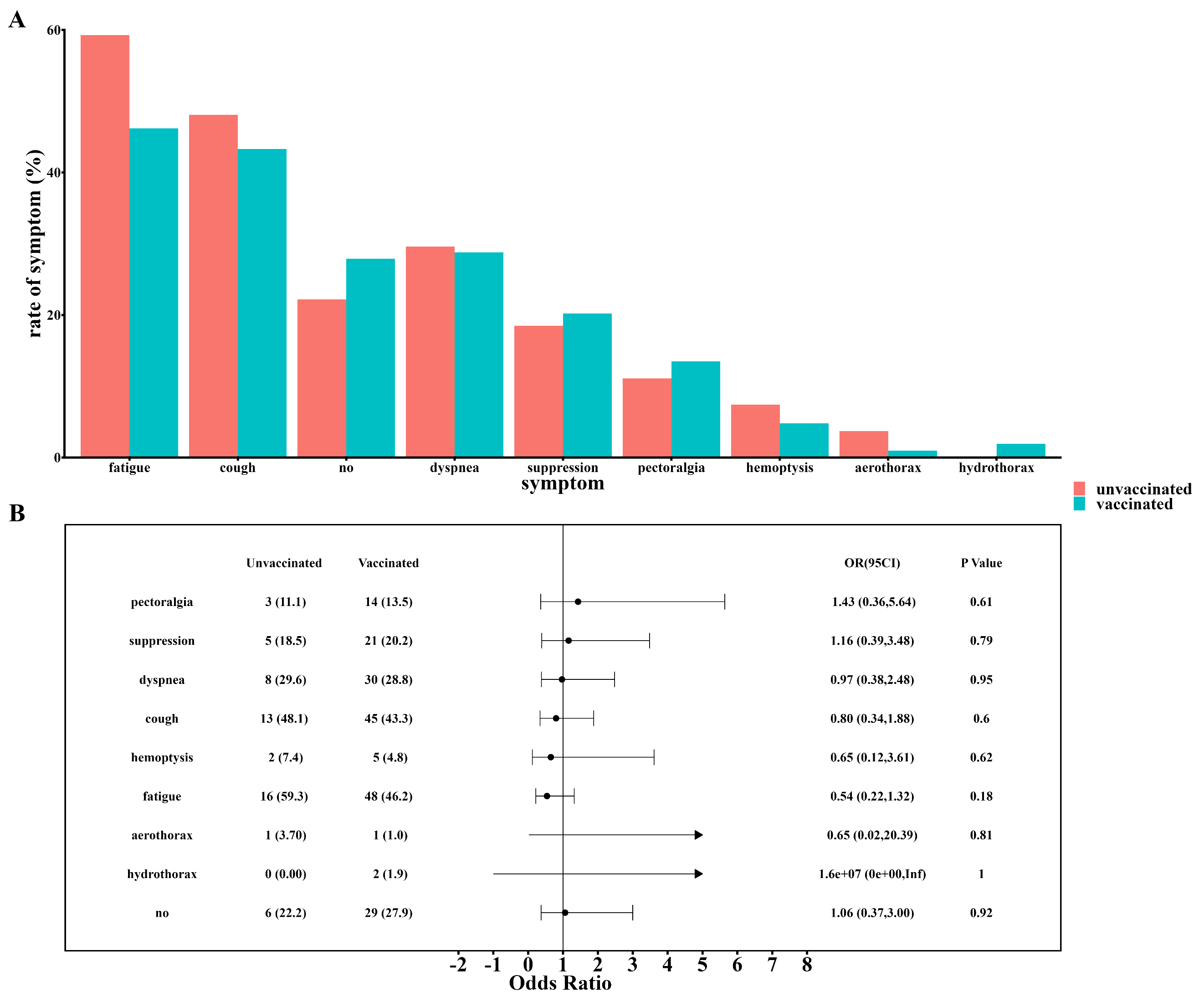


**Additional Figure 4. analysis of the risk of symptom exacerbation in vaccinated versus unvaccinated patients with LAM (n=131)**
