# Supplementary material for: Safety and efficacy of COVID-19 vaccination in the Chinese population with pulmonary lymphangioleiomyomatosis: a single-center retrospective study
Source: Orphanet J Rare Dis. 2024 Jul 3;19:247. doi: 10.1186/s13023-024-03260-4 (PMC11220960; doi:10.1186/s13023-024-03260-4)
Supplement: Supplementary file 5 — Supplementary Material 5 [file 13023_2024_3260_MOESM5_ESM.docx]

**Additional file 5: Logistic regression modeling of the risk of adverse reactions in vaccinated patients lam patients versus healthy adults**

ache

| **Covariate** | **OR(95%CI)** | **p-value** | **Global p-value** |
| --- | --- | --- | --- |
| **lam** |  |  | 0·22 |
| No | reference |  |  |
| Yes | 1·84 (0·69,4·91) |  |  |
| **Age** |  |  | 0·85 |
| 19~40 | reference |  |  |
| 40~60 | 1·14 (0·41,3·14) | 0·8 |  |
| $>$=60 | 2·13 (0·15,30·01) | 0·58 |  |
| **underlying disease** |  |  | 0·42 |
| No | reference |  |  |
| Yes | 0·48 (0·08,2·86) |  |  |
| **Vaccine dose** |  |  | **0·024** |
| 1dose | reference |  |  |
| 2dose | 0·15 (0·02,1·29) | 0·084 |  |
| 3dose | 0·24 (0·04,1·32) | 0·099 |  |
| 4dose | 1·64 (0·18,14·99) | 0·66 |  |

muscle

| **Covariate** | **OR(95%CI)** | **p-value** | **Global p-value** |
| --- | --- | --- | --- |
| **lam** |  |  | 0·69 |
| No | reference |  |  |
| Yes | 1·21 (0·47,3·11) |  |  |
| **Age cat** |  |  | 0·17 |
| 19~40 | reference |  |  |
| 40~60 | 2·08 (0·65,6·59) | 0·21 |  |
| $>$=60 | 5·48 (0·88,34·16) | 0·069 |  |

inflamed

| **Covariate** | **OR(95%CI)** | **p-value** | **Global p-value** |
| --- | --- | --- | --- |
| **lam** |  |  | 0·39 |
| No | reference |  |  |
| Yes | 0·45 (0·07,2·8) |  |  |
| **Age cat** |  |  | 0·99 |
| 19~40 | reference |  |  |
| 40~60 | 0·9 (0·23,3·46) | 0·87 |  |
| $>$=60 | 8·2e-07 (0e+00,Inf) | 1 |  |
| **BMI cat** |  |  | **0·035** |
| $<$18·5 | reference |  |  |
| 18 5~23 9 | 0·09 (0·01,0·67) | **0·018** |  |
| $>$=23 9 | 0·36 (0·04,2·96) | 0·34 |  |
| **hypertension** |  |  | 0·99 |
| No | reference |  |  |
| Yes | 1·6e-07 (0e+00,Inf) |  |  |
| **vaccine type** |  |  | **0·0055** |
| vero_only | reference |  |  |
| other | 18·42 (2·35,144·36) |  |  |

Induration

| **Covariate** | **OR(95%CI)** | **p-value** | **Global p-value** |
| --- | --- | --- | --- |
| **lam** |  |  | 0·61 |
| No | reference |  |  |
| Yes | 0·69 (0·16,2·96) |  |  |
| **Age cat** |  |  | 0·42 |
| 19~40 | reference |  |  |
| 40~60 | 4·15 (0·5,34·47) | 0·19 |  |
| $>$=60 | 8·7e-07 (0e+00,Inf) | 0·99 |  |

fever

| **Covariate** | **OR(95%CI)** | **p-value** | **Global p-value** |
| --- | --- | --- | --- |
| **lam** |  |  | 0·95 |
| No | reference |  |  |
| Yes | 1·05 (0·21,5·37) |  |  |
| **Age cat** |  |  | 0·98 |
| 19~40 | reference |  |  |
| 40~60 | 1·21 (0·22,6·82) | 0·83 |  |
| $>$=60 | 4·3e-07 (0e+00,Inf) | 0·99 |  |

fatigue

| **Covariate** | **OR(95%CI)** | **p-value** | **Global p-value** |
| --- | --- | --- | --- |
| **lam** |  |  | 0·91 |
| No | reference |  |  |
| Yes | 0·93 (0·27,3·16) |  |  |
| **Age cat** |  |  | 0·86 |
| 19~40 | reference |  |  |
| 40~60 | 1·38 (0·44,4·32) | 0·58 |  |
| $>$=60 | 6·1e-07 (0e+00,Inf) | 0·99 |  |
| **BMI cat** |  |  | 0·082 |
| $<$18·5 | reference |  |  |
| 18 5~23 9 | 0·2 (0·05,0·85) | **0·029** |  |
| $>$=23 9 | 0·38 (0·07,2·12) | 0·27 |  |

headache

| **Covariate** | **OR(95%CI)** | **p-value** | **Global p-value** |
| --- | --- | --- | --- |
| **lam** |  |  | 0·56 |
| No | reference |  |  |
| Yes | 0·6 (0·1,3·48) |  |  |
| **Age cat** |  |  | 0·8 |
| 19~40 | reference |  |  |
| 40~60 | 1·85 (0·3,11·31) | 0·51 |  |
| $>$=60 | 8·2e-08 (0e+00,Inf) | 1 |  |
| **Vaccine dose** |  |  | **0·043** |
| 1dose | reference |  |  |
| 2dose | 9·2e-09 (0e+00,Inf) | 0·99 |  |
| 3dose | 0·11 (0·01,1·16) | 0·066 |  |
| 4dose | 1·34 (0·08,21·88) | 0·84 |  |

arthralgia

| **Covariate** | **OR(95%CI)** | **p-value** | **Global p-value** |
| --- | --- | --- | --- |
| **lam** |  |  | 0·27 |
| No | reference |  |  |
| Yes | 0·26 (0·02,2·89) |  |  |
| **Age cat** |  |  | 0·99 |
| 19~40 | reference |  |  |
| 40~60 | 1·22 (0·11,13·47) | 0·87 |  |
| $>$=60 | 2·7e-07 (0e+00,Inf) | 0·99 |  |
| **underlying disease** |  |  | 0·28 |
| No | reference |  |  |
| Yes | 4·09 (0·32,51·83) |  |  |

Any adverse

| **Covariate** | **OR(95%CI)** | **p-value** | **Global p-value** |
| --- | --- | --- | --- |
| **lam** |  |  | 0·87 |
| No | reference |  |  |
| Yes | 1·06 (0·53,2·09) |  |  |
| **Age cat** |  |  | 0·88 |
| 19~40 | reference |  |  |
| 40~60 | 0·99 (0·47,2·08) | 0·98 |  |
| $>$=60 | 1·47 (0·28,7·75) | 0·65 |  |
| **underlying disease** |  |  | 0·24 |
| No | reference |  |  |
| Yes | 1·79 (0·67,4·78) |  |  |
