# Supplementary material for: Safety and efficacy of COVID-19 vaccination in the Chinese population with pulmonary lymphangioleiomyomatosis: a single-center retrospective study
Source: Orphanet J Rare Dis. 2024 Jul 3;19:247. doi: 10.1186/s13023-024-03260-4 (PMC11220960; doi:10.1186/s13023-024-03260-4)
Supplement: Supplementary file 6 — Supplementary Material 6 [file 13023_2024_3260_MOESM6_ESM.docx]

Additional file 6: Logistic regression modeling of the risk of vaccinated versus unvaccinated COVID-19 symptoms among LAM patients

cough

| **Covariate** | **OR(95%CI)** | **p-value** | **Global p-value** |
| --- | --- | --- | --- |
| **vaccine** |  |  | 0.66 |
| No | reference |  |  |
| Yes | 1.3 (0.41,4.08) |  |  |
| **Age** |  |  | 0.83 |
| 19~40 | reference |  |  |
| 40~60 | 0.77 (0.3,2.01) | 0.6 |  |
| $>$=60 | 1.21 (0.12,11.76) | 0.87 |  |
| **BMI** |  |  | 0.74 |
| $<$18.5 | reference |  |  |
| 18 5~23 9 | 0.62 (0.19,2.08) | 0.44 |  |
| $>$=23 9 | 7.4e+06 (0e+00,Inf) | 0.99 |  |

hoarse throat

| **Covariate** | **OR(95%CI)** | **p-value** | **Global p-value** |
| --- | --- | --- | --- |
| **vaccine** |  |  | 0.62 |
| No | reference |  |  |
| Yes | 1.25 (0.52,3.01) |  |  |
| **Age** |  |  | 0.58 |
| 19~40 | reference |  |  |
| 40~60 | 0.76 (0.37,1.55) | 0.44 |  |
| $>$=60 | 0.47 (0.08,2.63) | 0.39 |  |

anosmia

| **Covariate** | **OR(95%CI)** | **p-value** | **Global p-value** |
| --- | --- | --- | --- |
| **vaccine** |  |  | 0.77 |
| No | reference |  |  |
| Yes | 1.15 (0.45,2.91) |  |  |
| **Age** |  |  | 0.66 |
| 19~40 | reference |  |  |
| 40~60 | 0.77 (0.35,1.72) | 0.52 |  |
| $>$=60 | 1.43 (0.25,8.04) | 0.69 |  |
| **Underlying disease** |  |  | 0.97 |
| No | reference |  |  |
| Yes | 1.02 (0.38,2.77) |  |  |

sore throat

| **Covariate** | **OR(95%CI)** | **p-value** | **Global p-value** |
| --- | --- | --- | --- |
| **vaccine** |  |  | 0.86 |
| No | reference |  |  |
| Yes | 0.92 (0.37,2.3) |  |  |
| **Age** |  |  | **0.011** |
| 19~40 | reference |  |  |
| 40~60 | 0.35 (0.16,0.74) | **0.0065** |  |
| $>$=60 | 2.07 (0.23,18.71) | 0.52 |  |

vomiting

| **Covariate** | **OR(95%CI)** | **p-value** | **Global p-value** |
| --- | --- | --- | --- |
| **vaccine** |  |  | 0.84 |
| No | reference |  |  |
| Yes | 0.87 (0.24,3.17) |  |  |
| **Age** |  |  | 0.088 |
| 19~40 | reference |  |  |
| 40~60 | 0.28 (0.08,0.93) | **0.038** |  |
| $>$=60 | 0.98 (0.12,7.8) | 0.98 |  |
| **BMI** |  |  | 0.75 |
| $<$18.5 | reference |  |  |
| 18 5~23 9 | 0.63 (0.19,2.09) | 0.45 |  |
| $>$=23 9 | 2.7e-08 (0e+00,Inf) | 0.99 |  |
| **Underlying disease** |  |  | 0.1 |
| No | reference |  |  |
| Yes | 3.06 (0.81,11.6) |  |  |

dyspnea

| **Covariate** | **OR(95%CI)** | **p-value** | **Global p-value** |
| --- | --- | --- | --- |
| **vaccine** |  |  | 0.41 |
| No | reference |  |  |
| Yes | 0.66 (0.25,1.75) |  |  |
| **Age** |  |  | 0.69 |
| 19~40 | reference |  |  |
| 40~60 | 1.47 (0.59,3.62) | 0.41 |  |
| $>$=60 | 1.14 (0.16,8.32) | 0.9 |  |
| **BMI** |  |  | **0.031** |
| $<$18.5 | reference |  |  |
| 18 5~23 9 | 0.28 (0.11,0.72) | **0.0087** |  |
| $>$=23 9 | 0.36 (0.08,1.53) | 0.17 |  |
| **高血压** |  |  | 0.18 |
| No | reference |  |  |
| Yes | 2.28 (0.69,7.51) |  |  |

diarrhea

| **Covariate** | **OR(95%CI)** | **p-value** | **Global p-value** |
| --- | --- | --- | --- |
| **vaccine** |  |  | 0.25 |
| No | reference |  |  |
| Yes | 0.57 (0.22,1.49) |  |  |
| **Age** |  |  | 0.11 |
| 19~40 | reference |  |  |
| 40~60 | 0.44 (0.17,1.14) | 0.091 |  |
| $>$=60 | 1.67 (0.27,10.15) | 0.58 |  |
| **高血压** |  |  | **0.047** |
| No | reference |  |  |
| Yes | 3.47 (1.02,11.89) |  |  |

sweats

| **Covariate** | **OR(95%CI)** | **p-value** | **Global p-value** |
| --- | --- | --- | --- |
| **vaccine** |  |  | 0.31 |
| No | reference |  |  |
| Yes | 0.59 (0.21,1.64) |  |  |
| **Age** |  |  | 0.81 |
| 19~40 | reference |  |  |
| 40~60 | 0.73 (0.28,1.9) | 0.52 |  |
| $>$=60 | 5e-08 (0e+00,Inf) | 0.99 |  |
| **Underlying disease** |  |  | 0.16 |
| No | reference |  |  |
| Yes | 2.23 (0.73,6.79) |  |  |

fatigue

| **Covariate** | **OR(95%CI)** | **p-value** | **Global p-value** |
| --- | --- | --- | --- |
| **vaccine** |  |  | 0.2 |
| No | reference |  |  |
| Yes | 0.51 (0.18,1.43) |  |  |
| **Age** |  |  | 0.67 |
| 19~40 | reference |  |  |
| 40~60 | 0.79 (0.36,1.72) | 0.55 |  |
| $>$=60 | 1.6 (0.26,9.65) | 0.61 |  |
| **BMI** |  |  | **0.018** |
| $<$18.5 | reference |  |  |
| 18 5~23 9 | 0.36 (0.13,1.01) | 0.052 |  |
| $>$=23 9 | 3.9 (0.41,36.6) | 0.23 |  |

chest pain

| **Covariate** | **OR(95%CI)** | **p-value** | **Global p-value** |
| --- | --- | --- | --- |
| **vaccine** |  |  | 0.15 |
| No | reference |  |  |
| Yes | 0.47 (0.17,1.31) |  |  |
| **Age** |  |  | 0.74 |
| 19~40 | reference |  |  |
| 40~60 | 1.45 (0.52,4.06) | 0.47 |  |
| $>$=60 | 1.82 (0.24,13.58) | 0.56 |  |
| **BMI** |  |  | **0.047** |
| $<$18.5 | reference |  |  |
| 18 5~23 9 | 0.27 (0.09,0.76) | **0.014** |  |
| $>$=23 9 | 0.35 (0.07,1.76) | 0.2 |  |
| **Underlying disease** |  |  | 0.15 |
| No | reference |  |  |
| Yes | 2.24 (0.74,6.75) |  |  |

fever

| **Covariate** | **OR(95%CI)** | **p-value** | **Global p-value** |
| --- | --- | --- | --- |
| **vaccine** |  |  | 0.28 |
| No | reference |  |  |
| Yes | 0.42 (0.09,2.05) |  |  |
| **Age** |  |  | 0.19 |
| 19~40 | reference |  |  |
| 40~60 | 0.67 (0.23,1.9) | 0.45 |  |
| $>$=60 | 0.18 (0.03,1.14) | 0.069 |  |
| **BMI** |  |  | 0.48 |
| $<$18.5 | reference |  |  |
| 18 5~23 9 | 0.38 (0.08,1.81) | 0.22 |  |
| $>$=23 9 | 1.1e+07 (0e+00,Inf) | 0.99 |  |

headache

| **Covariate** | **OR(95%CI)** | **p-value** | **Global p-value** |
| --- | --- | --- | --- |
| **vaccine** |  |  | 0.057 |
| No | reference |  |  |
| Yes | 0.4 (0.15,1.03) |  |  |
| **Age** |  |  | 0.78 |
| 19~40 | reference |  |  |
| 40~60 | 1.05 (0.51,2.17) | 0.89 |  |
| $>$=60 | 1.88 (0.33,10.77) | 0.48 |  |

anosmia

| **Covariate** | **OR(95%CI)** | **p-value** | **Global p-value** |
| --- | --- | --- | --- |
| **vaccine** |  |  | **0.019** |
| No | reference |  |  |
| Yes | 0.34 (0.14,0.84) |  |  |
| **Age** |  |  | 0.65 |
| 19~40 | reference |  |  |
| 40~60 | 1.02 (0.44,2.36) | 0.96 |  |
| $>$=60 | 2.15 (0.41,11.32) | 0.37 |  |

muscle pain

| **Covariate** | **OR(95%CI)** | **p-value** | **Global p-value** |
| --- | --- | --- | --- |
| **vaccine** |  |  | **0.019** |
| No | reference |  |  |
| Yes | 0.34 (0.13,0.84) |  |  |
| **Age** |  |  | 0.95 |
| 19~40 | reference |  |  |
| 40~60 | 1.01 (0.49,2.09) | 0.97 |  |
| $>$=60 | 1.29 (0.25,6.54) | 0.76 |  |

anorexia

| **Covariate** | **OR(95%CI)** | **p-value** | **Global p-value** |
| --- | --- | --- | --- |
| **vaccine** |  |  | $<$**0.001** |
| No | reference |  |  |
| Yes | 0.17 (0.07,0.43) |  |  |
| **Age** |  |  | 0.14 |
| 19~40 | reference |  |  |
| 40~60 | 2.24 (0.93,5.4) | 0.072 |  |
| $>$=60 | 3.75 (0.59,23.68) | 0.16 |  |
| **Underlying disease** |  |  | 0.57 |
| No | reference |  |  |
| Yes | 1.34 (0.5,3.61) |  |  |

other symptom

| **Covariate** | **OR(95%CI)** | **p-value** | **Global p-value** |
| --- | --- | --- | --- |
| **vaccine** |  |  | 0.19 |
| No | reference |  |  |
| Yes | 0.32 (0.06,1.75) |  |  |
| **Age** |  |  | 0.056 |
| 19~40 | reference |  |  |
| 40~60 | 0.65 (0.1,4.18) | 0.65 |  |
| $>$=60 | 19.4 (1.2,314.73) | **0.037** |  |
| **Underlying disease** |  |  | 0.24 |
| No | reference |  |  |
| Yes | 0.17 (8.8e-03,3.23) |  |  |
