# Supplementary material for: Safety and efficacy of COVID-19 vaccination in the Chinese population with pulmonary lymphangioleiomyomatosis: a single-center retrospective study
Source: Orphanet J Rare Dis. 2024 Jul 3;19:247. doi: 10.1186/s13023-024-03260-4 (PMC11220960; doi:10.1186/s13023-024-03260-4)
Supplement: Supplementary file 7 — Supplementary Material 7 [file 13023_2024_3260_MOESM7_ESM.docx]

Additional file 7: COVID-19 for mTOR medication versus no mTOR medication among LAM patients vaccinated with inactivated vaccine Logistic regression modeling of the risk of symptom onset

fatigue

| **Covariate** | **OR(95%CI)** | **p-value** | **Global p-value** |
| --- | --- | --- | --- |
| **sirolimus everolimus** |  |  | **0.043** |
| No | reference |  |  |
| Yes | 0.18 (0.03,0.95) |  |  |
| **Age** |  |  | 0.21 |
| 19~40 | reference |  |  |
| 40~60 | 0.52 (0.2,1.33) | 0.17 |  |
| $>$=60 | 2.71 (0.25,29.25) | 0.41 |  |
| **BMI** |  |  | **0.045** |
| $<$18.5 | reference |  |  |
| 18 5~23 9 | 0.27 (0.08,0.94) | **0.039** |  |
| $>$=23 9 | 1.59 (0.14,18.16) | 0.71 |  |

fever

| **Covariate** | **OR(95%CI)** | **p-value** | **Global p-value** |
| --- | --- | --- | --- |
| **sirolimus everolimus** |  |  | 0.39 |
| No | reference |  |  |
| Yes | 0.38 (0.04,3.36) |  |  |
| **Age** |  |  | 0.093 |
| 19~40 | reference |  |  |
| 40~60 | 0.54 (0.16,1.77) | 0.31 |  |
| $>$=60 | 0.07 (5.7e-03,0.79) | **0.032** |  |
| **BMI** |  |  | 0.7 |
| $<$18.5 | reference |  |  |
| 18 5~23 9 | 0.5 (0.1,2.49) | 0.4 |  |
| $>$=23 9 | 4e+06 (0e+00,Inf) | 0.99 |  |

cough

| **Covariate** | **OR(95%CI)** | **p-value** | **Global p-value** |
| --- | --- | --- | --- |
| **sirolimus everolimus** |  |  | 0.56 |
| No | reference |  |  |
| Yes | 0.62 (0.12,3.11) |  |  |
| **Age** |  |  | 0.4 |
| 19~40 | reference |  |  |
| 40~60 | 0.46 (0.14,1.45) | 0.19 |  |
| $>$=60 | 0.44 (0.04,5.15) | 0.51 |  |

anorexia

| **Covariate** | **OR(95%CI)** | **p-value** | **Global p-value** |
| --- | --- | --- | --- |
| **sirolimus everolimus** |  |  | **0.043** |
| No | reference |  |  |
| Yes | 0.3 (0.09,0.96) |  |  |
| **Age** |  |  | 0.34 |
| 19~40 | reference |  |  |
| 40~60 | 2.16 (0.76,6.13) | 0.15 |  |
| $>$=60 | 2.06 (0.18,23.34) | 0.56 |  |

dyspnea

| **Covariate** | **OR(95%CI)** | **p-value** | **Global p-value** |
| --- | --- | --- | --- |
| **sirolimus everolimus** |  |  | 0.67 |
| No | reference |  |  |
| Yes | 1.38 (0.32,6.02) |  |  |
| **Age** |  |  | 0.63 |
| 19~40 | reference |  |  |
| 40~60 | 1.36 (0.44,4.23) | 0.6 |  |
| $>$=60 | 3.18 (0.3,34.04) | 0.34 |  |
| **BMI** |  |  | 0.056 |
| $<$18.5 | reference |  |  |
| 18 5~23 9 | 0.24 (0.07,0.77) | **0.017** |  |
| $>$=23 9 | 0.32 (0.05,2.2) | 0.24 |  |
| **并发症** |  |  | 0.2 |
| No | reference |  |  |
| Yes | 2.31 (0.65,8.19) |  |  |

headache

| **Covariate** | **OR(95%CI)** | **p-value** | **Global p-value** |
| --- | --- | --- | --- |
| **sirolimus everolimus** |  |  | 0.54 |
| No | reference |  |  |
| Yes | 0.69 (0.21,2.3) |  |  |
| **Age** |  |  | 0.98 |
| 19~40 | reference |  |  |
| 40~60 | 1.02 (0.42,2.47) | 0.97 |  |
| $>$=60 | 0.83 (0.1,6.7) | 0.86 |  |
| **Vaccine dose** |  |  | 0.35 |
| unvaccinated | reference |  |  |
| 2dose | 1.5e+07 (0e+00,Inf) | 0.99 |  |
| 3dose | 7.1e+06 (0e+00,Inf) | 0.99 |  |

ageusia

| **Covariate** | **OR(95%CI)** | **p-value** | **Global p-value** |
| --- | --- | --- | --- |
| **sirolimus everolimus** |  |  | **0.0093** |
| No | reference |  |  |
| Yes | 0.2 (0.06,0.67) |  |  |
| **Age** |  |  | 0.74 |
| 19~40 | reference |  |  |
| 40~60 | 1.37 (0.45,4.12) | 0.58 |  |
| $>$=60 | 2.27 (0.2,26.01) | 0.51 |  |

anosmia

| **Covariate** | **OR(95%CI)** | **p-value** | **Global p-value** |
| --- | --- | --- | --- |
| **sirolimus everolimus** |  |  | 0.24 |
| No | reference |  |  |
| Yes | 0.49 (0.15,1.6) |  |  |
| **Age** |  |  | 0.64 |
| 19~40 | reference |  |  |
| 40~60 | 0.83 (0.32,2.14) | 0.71 |  |
| $>$=60 | 2.27 (0.27,19.18) | 0.45 |  |
| **Vaccine dose** |  |  | 0.11 |
| unvaccinated | reference |  |  |
| 2dose | 1.3 (0.07,25.76) | 0.86 |  |
| 3dose | 0.44 (0.02,8.22) | 0.58 |  |

diarrhea

| **Covariate** | **OR(95%CI)** | **p-value** | **Global p-value** |
| --- | --- | --- | --- |
| **sirolimus everolimus** |  |  | 0.22 |
| No | reference |  |  |
| Yes | 0.44 (0.12,1.62) |  |  |
| **Age** |  |  | 0.35 |
| 19~40 | reference |  |  |
| 40~60 | 0.73 (0.25,2.15) | 0.57 |  |
| $>$=60 | 3.66 (0.41,32.4) | 0.24 |  |
| **Vaccine dose** |  |  | NA |
| unvaccinated | reference |  |  |
| 2dose | 1.5e+07 (0e+00,Inf) | 0.99 |  |
| 3dose | 4.4e+06 (0e+00,Inf) | 0.99 |  |

vomiting

| **Covariate** | **OR(95%CI)** | **p-value** | **Global p-value** |
| --- | --- | --- | --- |
| **sirolimus everolimus** |  |  | 0.55 |
| No | reference |  |  |
| Yes | 0.65 (0.15,2.74) |  |  |
| **Age** |  |  | 0.29 |
| 19~40 | reference |  |  |
| 40~60 | 0.4 (0.12,1.33) | 0.14 |  |
| $>$=60 | 1.27 (0.12,13.95) | 0.84 |  |

chest pain

| **Covariate** | **OR(95%CI)** | **p-value** | **Global p-value** |
| --- | --- | --- | --- |
| **sirolimus everolimus** |  |  | 0.055 |
| No | reference |  |  |
| Yes | 0.28 (0.08,1.03) |  |  |
| **Age** |  |  | 0.59 |
| 19~40 | reference |  |  |
| 40~60 | 1.59 (0.47,5.33) | 0.45 |  |
| $>$=60 | 3.14 (0.26,37.53) | 0.37 |  |

sweats

| **Covariate** | **OR(95%CI)** | **p-value** | **Global p-value** |
| --- | --- | --- | --- |
| **sirolimus everolimus** |  |  | 0.74 |
| No | reference |  |  |
| Yes | 0.79 (0.19,3.24) |  |  |
| **Age** |  |  | 0.88 |
| 19~40 | reference |  |  |
| 40~60 | 1.33 (0.43,4.13) | 0.62 |  |
| $>$=60 | 1.4e-07 (0e+00,Inf) | 0.99 |  |

muscle pain

| **Covariate** | **OR(95%CI)** | **p-value** | **Global p-value** |
| --- | --- | --- | --- |
| **sirolimus everolimus** |  |  | 0.91 |
| No | reference |  |  |
| Yes | 0.94 (0.31,2.86) |  |  |
| **Age** |  |  | 0.82 |
| 19~40 | reference |  |  |
| 40~60 | 1.3 (0.56,3.01) | 0.55 |  |
| $>$=60 | 1.43 (0.18,11.21) | 0.74 |  |

sore throat

| **Covariate** | **OR(95%CI)** | **p-value** | **Global p-value** |
| --- | --- | --- | --- |
| **sirolimus everolimus** |  |  | 0.49 |
| No | reference |  |  |
| Yes | 0.65 (0.19,2.2) |  |  |
| **Age** |  |  | **0.024** |
| 19~40 | reference |  |  |
| 40~60 | 0.29 (0.12,0.72) | **0.0081** |  |
| $>$=60 | 1.02 (0.1,11.04) | 0.98 |  |

Hoarse throat

| **Covariate** | **OR(95%CI)** | **p-value** | **Global p-value** |
| --- | --- | --- | --- |
| **sirolimus everolimus** |  |  | 0.92 |
| No | reference |  |  |
| Yes | 0.94 (0.31,2.89) |  |  |
| **Age** |  |  | 0.66 |
| 19~40 | reference |  |  |
| 40~60 | 0.68 (0.29,1.57) | 0.37 |  |
| $>$=60 | 0.96 (0.12,7.54) | 0.97 |  |
